# Antibiotic-induced perturbations in gut microbial diversity influences neuro-inflammation and amyloidosis in a murine model of Alzheimer’s disease

**DOI:** 10.1038/srep30028

**Published:** 2016-07-21

**Authors:** Myles R. Minter, Can Zhang, Vanessa Leone, Daina L. Ringus, Xiaoqiong Zhang, Paul Oyler-Castrillo, Mark W. Musch, Fan Liao, Joseph F. Ward, David M. Holtzman, Eugene B. Chang, Rudolph E. Tanzi, Sangram S. Sisodia

**Affiliations:** 1 Department of Neurobiology, The University of Chicago, Chicago, IL, 60637, USA; 2 The Microbiome Center, The University of Chicago, Chicago, IL, 60637, USA; 3 Department of Neurology, Genetics and Aging Research Unit, MassGeneral Institute for Neurodegenerative Diseases, Massachusetts General Hospital, Charlestown, MA 02114, USA; 4 Department of Medicine, The University of Chicago, Chicago, IL, 60637, USA; 5 Department of Neurology, Hope Center for Neurological Disorders, Charles F. and Joanne Knight Alzheimer’s Disease Research Center, Washington University School of Medicine, St. Louis, MO, 63110, USA

## Abstract

Severe amyloidosis and plaque-localized neuro-inflammation are key pathological features of Alzheimer’s disease (AD). In addition to astrocyte and microglial reactivity, emerging evidence suggests a role of gut microbiota in regulating innate immunity and influencing brain function. Here, we examine the role of the host microbiome in regulating amyloidosis in the APP_SWE_/PS1_ΔE9_ mouse model of AD. We show that prolonged shifts in gut microbial composition and diversity induced by long-term broad-spectrum combinatorial antibiotic treatment regime decreases Aβ plaque deposition. We also show that levels of soluble Aβ are elevated and that levels of circulating cytokine and chemokine signatures are altered in this setting. Finally, we observe attenuated plaque-localised glial reactivity in these mice and significantly altered microglial morphology. These findings suggest the gut microbiota community diversity can regulate host innate immunity mechanisms that impact Aβ amyloidosis.

Alzheimer’s disease (AD), a progressive neurodegenerative disorder, is characterized by the presence of extracellular aggregates of amyloid-β (Aβ) peptides and intraneuronal neurofibrillary tangles (NFT). Neuro-inflammation is also a consistent pathological feature of AD^1^,^2^,^3^; PET scans of reactive microglia in human patients using [C^11^] R-PK11195 and [C^11^] DAA1106 tracers revealed a strong correlation between microgliosis, neuro-inflammatory burden and progressive cognitive decline^4^,^5^. While it is widely agreed that Aβ plays a central role in initiating disease, it is perhaps the severity of neuro-inflammation that regulates progression of cognitive decline. In support of this notion, recent GWAS studies have identified numerous genetic risk factors for late-onset AD that are associated with innate immunity, including *CD33* and *TREM2*, that encode molecules important in microglial phagocytosis and clearance of Aβ^6^,^7^,^8^,^9^,^10^,^11^.

Soluble Aβ peptides are detected by the toll-like receptors (TLRs), a super-family of pattern recognition receptors, which are expressed by microglia and induce activation of inflammasome complexes leading to initiation of neuro-inflammatory responses^12^. Based on the activation stimulus and environmental niche, these CNS-residing immune cells can display pro-inflammatory or anti-inflammatory and immune resolving activity. In this regard, the mechanisms that control microglial polarization necessary for promoting the clearance of Aβ from the brain^13^,^14^,^15^,^16^,^17^ are still unclear. However, recent studies have demonstrated that peripheral immune components also play a major role in CNS neuro-inflammatory responses. Specifically, type-1 interferon responses from peripheral T-cell populations recruited to the CNS across the blood-CSF barrier accelerate many of the deleterious signs of aging^18^ with type-1 interferon signaling being implicated in AD^19^. Indeed, transient depletion of peripheral Foxp3^+^ regulatory T-cells re-balances innate neuro-inflammatory responses and alleviates amyloidosis in 5xFAD mice^20^. Thus, while it is known that peripheral immune challenges trigger centralized neuro-inflammatory responses^21^, the regulatory mechanisms that govern the propensity to initiate these responses in AD remains largely unknown.

Over the past decade, it has become apparent that gut microbes play a critical role in the regulation of brain function via homeostatic control of host innate immunity^22^. During vaginal delivery, the gastrointestinal tract of the newborn is colonized by the microbiota in the maternal genital tract and differs significantly from newborns delivered by caesarian section. Human neonates born by caesarian section display less complex brain electrical activity than those born vaginally^23^ and cesearian-delivered rats exhibit alterations in pre-pubertal prefrontal cortex and hippocampus development^24^. Intestinal microbes release microbe-associated molecular patterns (MAMPs) that are detected by TLRs^25^,^26^. For example, polysaccharide A (PSA), a symbiosis factor produced by the human intestinal commensal *Bacteroides fragilis*, protects against CNS demyelination and inflammation during experimental autoimmune encephalomyelitis (EAE). PSA signals through TLR2, mediating expansion of a critical regulatory CD39^+^ CD4 T-cell subset. Mice lacking *TLR2* or *CD39* abrogates PSA-mediated CNS-targeted protection of EAE-mediated demyelination^27^. A recent study highlights the role of gut microbiota in regulating microglial maturation and function in mice. Microglia isolated from germ-free mice display significantly altered inflammatory gene expression profiles that influence the basal surveillance (M0) state of these cells, altered cellular morphology and attenuated, but not ablated, inflammatory responses to bacterial and viral challenge. This phenotype is largely mimicked by antibiotic administration to deplete gut microbiota and can be restored by supplementation with microbially-derived metabolites, specifically short-chain fatty acids^28^. These studies highlight a complex role of gut microbes in regulating innate immunity and brain function.

Given the body of evidence suggesting a significant role for gut microbes in controlling host immunity and brain function we hypothesized that the composition of the intestinal microbiota plays a key role in modulating neuro-inflammation that in turn, influences Aβ deposition. In this observational study, we demonstrate that long-term antibiotic-treated male APP_SWE_/PS1_ΔE9_ mice display altered composition of the gut microbiota and peripheral inflammatory milieu that coincides with attenuated amyloid plaque pathology. In addition we observed elevated soluble Aβ1-40 and Aβ1-42 levels in these mice alongside attenuated plaque-localised microglial and astrocyte reactivity. Our findings suggest that persistent gut microbial dysbiosis regulates host innate immunity mechanisms that impacts Aβ amyloidosis.

## Results

### ABX-treated APP_SWE_/PS1_ΔE9_ mice display alterations in gastrointestinal microbiome composition and circulating inflammatory mediators

**Figure 1:**
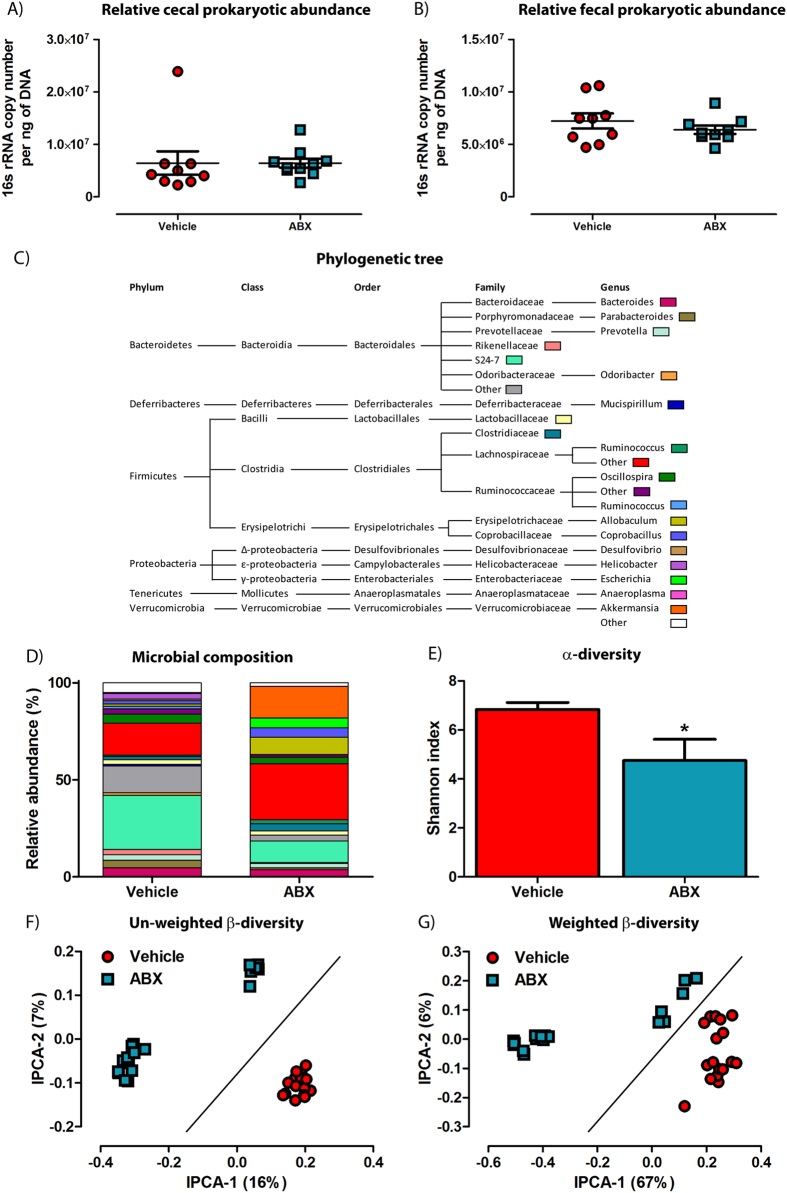
Composition of the gastrointestinal microbiome is altered in ABX-treated APP_SWE_/PS1_ΔE9_ mice. (**A**) Q-PCR analysis of isolated DNA from cecal contents of 6 month old male APP_SWE_/PS1_ΔE9_ mice analysing 16s rRNA gene copy number (*n* = 9–10). (**B**) Q-PCR analysis of isolated DNA from fecal contents of 6 month old male APP_SWE_/PS1_ΔE9_ mice analysing 16s rRNA gene copy number (*n* = 9–10). To obtain a 16s rRNA gene copy number value, expression levels were normalized to both DNA concentration and an amplification standard curve of a 16s rRNA gene-containing plasmid with known copy number. Illumina® MiSeq based sequencing of the 16s rRNA gene from cecal and fecal contents of 6 month old male APP_SWE_/PS1_ΔE9_ mice was performed and a microbial (**C**) phylogenetic tree and (**D**) diversity histogram were generated based on quality-controlled OTU reads. Only families and genus’ with relative abundance >0.5% were included. (**E**) α-diversity analysis of the sequencing data using the Shannon index, reveals a significant decrease in microbial diversification in ABX-treated APP_SWE_/PS1_ΔE9_ mice (*n* = 9–10, *p = 0.0429, unpaired two-tailed Student’s *t*-test). Principal co-ordinate analysis of (**F**) un-weighted, accounting for presence of OTUs only, and (**G**) weighted β-diversity, accounting for both presence and relative abundance of OTUs, demonstrates distinct alterations in microbial diversity in ABX-treated APP_SWE_/PS1_ΔE9_ mice (*n* = 9–10 mice, each with cecal and fecal sequencing). The percentage of data variance explained by each IPCA is displayed. Data are displayed as X/Y scatter or mean ± SEM. See Supplementary Figure 2–3, statistical Table 1 and Supplementary table 2 for additional information.

To examine the impact of the gut microbes on amyloid deposition, we treated 2-week old APP_SWE_/PS1_ΔE9_ mice^29^,^30^ with a high dose cocktail of antibiotics (ABX; composition in methods) or PBS vehicle by daily oral gavage for 1 week, then placed ABX-gavaged animals on a chronic dose (1/50^th^) of antibiotics in the drinking water for the remainder of the experiment. To ascertain if ABX treatment affects total microbial abundance at the time of cull, DNA isolated from cecal and fecal contents was subjected to quantitative PCR (Q-PCR). Surprisingly, analysis of 16s rRNA gene copy number revealed no change between ABX-treated APP_SWE_/PS1_ΔE9_ mice and vehicle controls at the time of cull (Cecal: 4.3×10^^6^ ± 5.4 × 10^^5^ vs. 6.4 × 10^^6^ ± 8.6 × 10^^5^ copies/ng DNA; Fecal: 7.2 × 10^^6^ ± 7.1 × 10^^5^ vs. 6.4 × 10^^6^ ± 4.0 × 10^^5^ copies/ng DNA; *n* = 9–10; Fig. 1A,B). Despite observing no difference in total microbial 16S rRNA gene abundance, we next determined if ABX treatment did indeed have an impact on effectively eliminating gut microbes. Here, we obtained and cultured bacteria from fecal homogenates of ABX and vehicle treated male APP_SWE_/PS1_ΔE9_ mice in LB media in the presence of our ABX cocktail. We confirmed that while cultures from vehicle-treated animals were susceptible to ABX-induced toxicity, cultures derived from ABX-treated mice were largely resistant (0.071 ± 0.00093 vs. 0.65 ± 0.0057, O.D._600nm_, p < 0.0001, *n* = 6; Supp. Fig. 3). Thus the bacterial load in feces of ABX-treated animals represented significantly altered bacterial populations as compared to those present in vehicle control mice.

To characterize the effect of chronic ABX treatment on the gastrointestinal microbial composition in APP_SWE_/PS1_ΔE9_ mice, we amplified the 16s rRNA gene from DNA isolated from cecal and fecal contents taken at the time of cull and performed terminal restriction fragment length polymorphism (T-RFLP) analysis. Analysis of 16s rRNA fragment size and grouping by principal component analysis (PCA) displays a difference in the clustering of fragments obtained from male ABX-treated APP_SWE_/PS1_ΔE9_ mice compared to vehicle control (Supp. Fig. 2A,B). A similar diversification was likewise identified from T-RFLP analysis of cecal and fecal 16s rRNA gene DNA in female APP_SWE_/PS1_ΔE9_ mice (Supp. Fig. 2C,D).

We next employed Illumina® MiSeq-based high throughput amplicon sequencing of the V4-V5 16S rRNA microbial gene to identify gut microbial community membership in cecal and fecal contents of ABX and vehicle treated male APP_SWE_/PS1_ΔE9_ mice. Using the QIIME, taxonomic classification analysis of operational taxonomic units (OTUs) derived from the primary sequencing data revealed altered microbial composition in ABX-treated APP_SWE_/PS1_ΔE9_ mice as compared to vehicle controls (*n* = 9–10, individual fecal and cecal reads, Fig. 1C,D, Supp. Table 2). Dramatic alterations in microbial community composition were observed in both feces and cecal contents. Interestingly we observed an expansion of the genus *Akkermansia* (0.022% vs. 16.46%) and family *Lachnospiraceae* (5.10% vs. 12.50%) in ABX-treated APP_SWE_/PS1_ΔE9_ mice (Fig. 1E,F, Supp. Table 2). We then assessed the microbial diversity within each treatment group (α-diversity) by calculating Shannon indices that assume all microbial species are represented in our samples and that random sampling occurs. Using this index, we confirm a significant decrease in microbial α-diversity in ABX-treated APP_SWE_/PS1_ΔE9_ mice (6.84 ± 0.28 vs. 4.75 ± 0.87, p = 0.0429, *n* = 9–10, Fig. 1E). We then assessed the dissimilarity of individual microbial communities between treatment groups (β-diversity). PCA plotting of un-weighted β-diversity dissimilarity matrix data, accounting for presence of OTUs but not abundance, revealed a distinct clustering effect in ABX-treated APP_SWE_/PS1_ΔE9_ mice compared to vehicle controls (*n* = 9–10, individual fecal and cecal reads, Fig. 1F), supporting our initial findings using T-RFLP (Supp. Fig. 2A,B). Examination of weighted β-diversity PCA dissimilarity matrix data, accounting for presence and abundance of OTUs, also revealed a similar distinct clustering effect in ABX-treated APP_SWE_/PS1_ΔE9_ mice compared to vehicle controls (*n* = 9–10, individual fecal and cecal reads, Fig. 1G). Collectively these data highlight that microbial diversity, but not total abundance, is altered by chronic ABX treatment in APP_SWE_/PS1_ΔE9_ mice.

**Figure 2:**
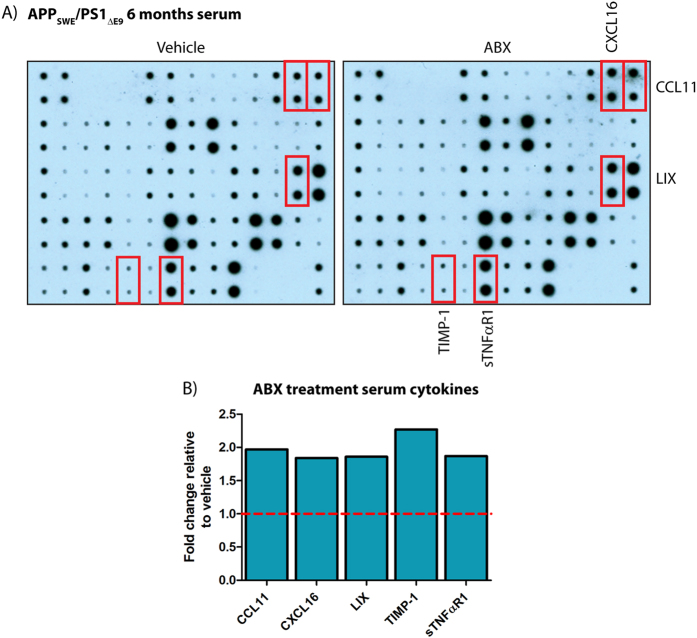
The circulating inflammatory mediator profile is altered in ABX-treated APP_SWE_/PS1_ΔE9_ mice. (**A**) Immunoblot-based array of inflammatory mediators in isolated serum from vehicle control and ABX-treated male 6 month old APP_SWE_/PS1_ΔE9_ mice (*n* = 10, pooled sera). (**B**) Densitometry of select inflammatory mediators from the aforementioned array. Expression levels in ABX-treated mice are expressed in arbitrary units relative to vehicle control (dashed line). Data are displayed as mean alone. See Supplementary Figures 4 and 5 for additional information.

Recent studies suggest that the peripheral innate immune system is regulated by the gastrointestinal microbial composition, innate ligands, and metabolites produced by these communities^31^,^32^,^33^. To assess if production of circulating chemokines and cytokines is altered by long-term ABX treatment in APP_SWE_/PS1_ΔE9_ mice, we probed serum from vehicle or ABX-treated mice with commercially available chemokine/cytokine arrays (Fig. 2A). Densitometric analysis of these arrays revealed numerous changes in the inflammatory profile of pooled sera isolated from 10 male ABX-treated APP_SWE_/PS1_ΔE9_ mice compared to vehicle controls (Supp. Fig. 4). Specifically up-regulations of CCL11 (1.97-fold), CXCL16 (1.84-fold), LIX (1.86-fold), TIMP-1 (2.27-fold) and NFαR1 (1.87-fold) were identified in male ABX-treated APP_SWE_/PS1_ΔE9_ mice (Fig. 2B). Interestingly these alterations in the inflammatory profile of male sera were not observed in female ABX-treated APP_SWE_/PS1_ΔE9_ mice (*n* = 8–10, Supp. Fig. 4). These findings suggest that circulating peripheral inflammatory mediator levels are altered in male ABX-treated APP_SWE_/PS1_ΔE9_ mice and supports the notion that microbe-derived signaling communicates with the host innate immune response.

To investigate a potential source of this altered systemic inflammatory profile, we isolated spleens and blood lymphocyte populations from vehicle and ABX-treated APP_SWE_/PS1_ΔE9_ mice and performed Q-PCR to analyze mRNAs encoding inflammatory mediators. While we detected a small but significant increase in splenic IFNβ levels in ABX-treated APP_SWE_/PS1_ΔE9_ mice, we observed no quantifiable difference between treatment groups in expression of IFIT-1, IRF-7, CCL11, IL-1β or TNFα in either spleen or lymphocyte preparations (Spleen: *n* = 5, lymphocytes: *n* = 3 each sample is pooled from 3 individual mice, Supp. Fig. 5A,B).

### Male ABX-treated APP_SWE_/PS1_ΔE9_ mice display reduced Aβ deposition but increased soluble Aβ levels

**Figure 3:**
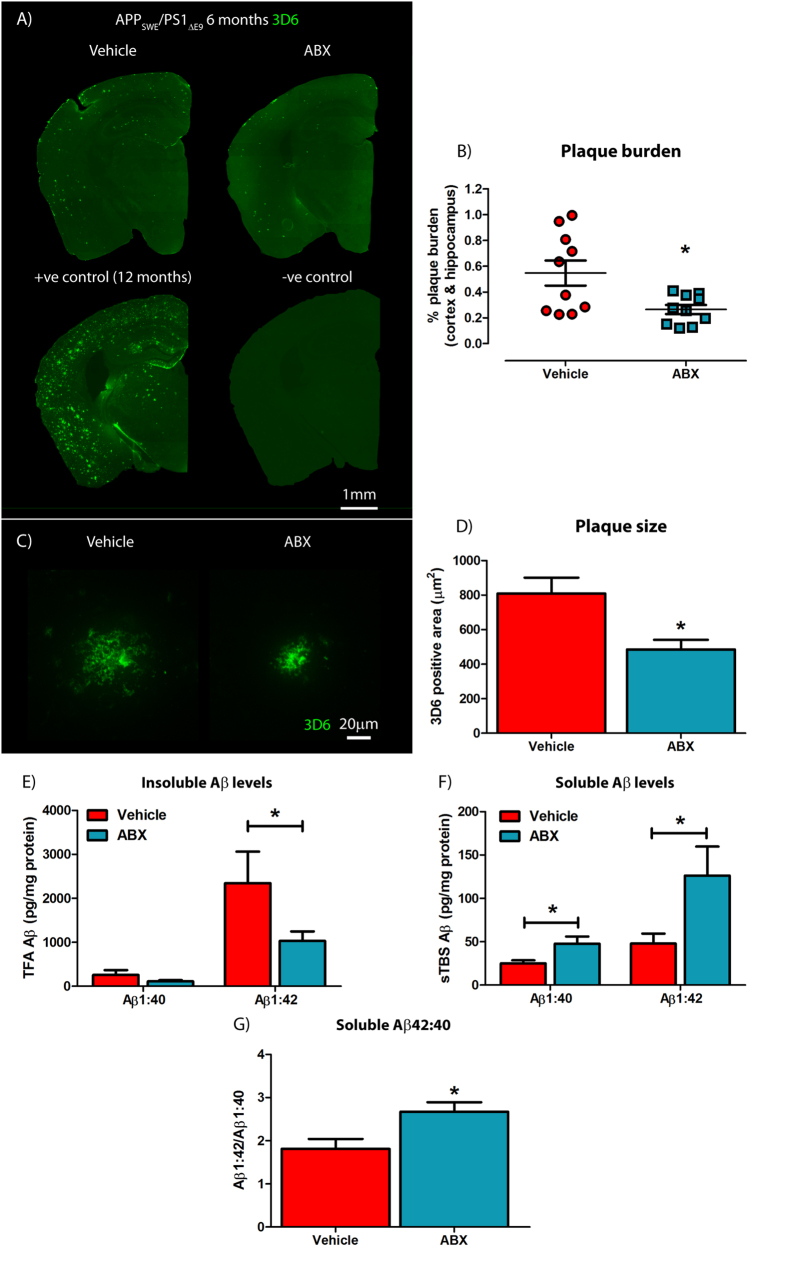
Amyloidosis is altered in ABX-treated male APP_SWE_/PS1_ΔE9_ mice. (**A**) Representative immunohistochemical images of Aβ plaque deposition in vehicle control and ABX-treated male 6 month old APP_SWE_/PS1_ΔE9_ mice using anti-Aβ mAb 3D6. Each staining run was performed using sections from 12 month old APP_SWE_/PS1_ΔE9_ mice as a positive control and no primary antibody negative controls. (**B**) Plaque burden quantification in vehicle control and ABX-treated male 6 month old APP_SWE_/PS1_ΔE9_ mice using particle analysis of 3D6^+ve^ immunofluorescence (*n* = 10, *p = 0.0169, unpaired two-tailed Student’s *t*-test). 3D6^+ve^ area was averaged from 4 sections/mouse (240 μm apart) and expressed relative to total cortical and hippocampal area. (**C**) Representative x60 magnification z-stack maximum projection images of 3D6^+ve^ Aβ plaques from vehicle control and ABX-treated male 6 month old APP_SWE_/PS1_ΔE9_ mice. (**D**) Quantification of plaque area using threshold-limiting immunofluorescence detection (*n* = 10, *p = 0.02, unpaired two-tailed Student’s *t*-test). (**E**) MSD Mesoscale® analysis of TFA-soluble (TBS-insoluble) Aβ1-40 and Aβ1-42 levels in combined cortical and hippocampal tissue of vehicle control and ABX-treated male 6 month old APP_SWE_/PS1_ΔE9_ mice using anti-Aβ mAb 4G8 (*n* = 10, *p = 0.0491, unpaired two-tailed Student’s *t*-test). (**F**) MSD Mesoscale® analysis of TBS-soluble Aβ1-40 and Aβ1-42 levels in combined cortical and hippocampal tissue of vehicle control and ABX-treated male 6 month old APP_SWE_/PS1_ΔE9_ mice using anti-Aβ mAb 4G8 (*n* = 10, *p < 0.05, unpaired two-tailed Student’s *t*-test). (**G**) Quantification of the Aβ1-40:Aβ1-42 ratio from the TBS-soluble MSD Mesoscale® data in vehicle control and ABX-treated male 6 month old APP_SWE_/PS1_ΔE9_ mice (*n* = 10, *p = 0.014, unpaired two-tailed Student’s *t*-test). Data are displayed as mean ± SEM. See Supplementary Figure 6–9 and statistical table 1 for additional information.

To investigate the impact of ABX-treatment on Aβ deposition in the brains of APP_SWE_/PS1_ΔE9_ mice, we sacrificed ABX-treated and vehicle-treated animals at 6 months of age, removed the brain and generated serial sections from each hemibrain. Sections were subjected to immunostaining with Aβ-specific 3D6 antibody^34^ and Aβ burden was quantified by stereology. These studies revealed a significant 2.27-fold decrease in combined cortical and hippocampal Aβ plaque burden in male ABX-treated APP_SWE_/PS1_ΔE9_ mice compared to vehicle controls (0.55 ± 0.097% vs. 0.24 ± 0.039%, *n* = 10, p = 0.0169, Fig. 3A,B). High power x60 magnification z-stack images of these Aβ plaques also revealed a significant 1.57-fold decrease in the size of individual deposits in male ABX-treated APP_SWE_/PS1_ΔE9_ mice compared to vehicle controls (762.8 ± 93.91 μm^2^ vs. 485.1 ± 56.18 μm^2^, *n* = 10, 4 plaques per mouse, p = 0.02, Fig. 3C,D). To validate these immunohistological findings, we removed the striatum from the opposite hemisphere and prepared TBS-soluble and TBS-insoluble fractions from the remaining tissue in order to determine levels of Aβ species using a MSD MesoScale® ELISA-based assay. We did not observe any differences in TBS-insoluble Aβ1-40 levels, but a significant 2.27-fold reduction in TBS-insoluble Aβ1-42 levels was apparent in male ABX-treated APP_SWE_/PS1_ΔE9_ mice compared to vehicle controls (2345.35 ± 717.08 vs. 1032.92 ± 215.01 pg/mg total protein, *n* = 10, p = 0.0491, Fig. 3E). Moreover, we observed a clear correlation between 3D6-positive Aβ plaque burden and TBS-Insoluble Aβ levels in both ABX-treated and vehicle control APP_SWE_/PS1_ΔE9_ mice, thus validating our assays (Supp. Fig. 6A–E). Collectively, these immunocytochemical and biochemical findings reveal that ABX treatment reduces Aβ plaque burden in young male APP_SWE_/PS1_ΔE9_ mice.

We also assessed the TBS-soluble fraction of these same tissues using the MSD MesoScale® assay. Interestingly, a significant 1.9-fold up-regulation in TBS-soluble Aβ1-40 and 2.63-fold up-regulation in TBS-soluble Aβ1-42 levels were detected in the brains of male ABX-treated APP_SWE_/PS1_ΔE9_ mice compared with vehicle controls (Aβ1-40: 25.07 ± 3.60 vs. 47.72 ± 8.32 pg/mg total protein, p = 0.0178; Aβ1-42: 47.94 ± 11.47 vs. 126.29 ± 33.53 pg/mg total protein, p = 0.0326, *n* = 10, Fig. 3F). Calculating the ratio of TBS-soluble Aβ1-42:TBS-soluble Aβ1-40 revealed that male ABX-treated APP_SWE_/PS1_ΔE9_ mice exhibit enhanced skewing towards Aβ1-42 production in comparison with vehicle controls (1.81 ± 0.23 vs. 2.67 ± 0.22, *n* = 10, p = 0.014, Fig. 3G). These findings demonstrate that ABX treatment enhances soluble Aβ production but restricts plaque deposition in male APP_SWE_/PS1_ΔE9_ mice.

We considered the possibility that our findings might be the result of decreased transgene expression in the brains of the APP_SWE_/PS1_ΔE9_ mice treated with ABX. To clarify this issue, we performed Western blot examination with human APP-specific 6E10 antibody followed by densitometric quantification. These studies revealed no differences in the levels of full length human APP expression in RIPA-soluble brain lysates between ABX-treated and vehicle control APP_SWE_/PS1_ΔE9_ mice (*n* = 7, Supp. Fig. 7A,B).

As alterations in brain ApoE concentrations can regulate production of soluble Aβ species and aggregation^35^,^36^,^37^, we quantified the levels of ApoE in the brains of ABX-treated male APP_SWE_/PS1_ΔE9_ mice by ELISA. These studies revealed no differences in brain ApoE levels between ABX-treated and vehicle control male APP_SWE_/PS1_ΔE9_ mice (*n* = 10, Supp. Fig. 8). Correlation analysis revealed no distinct relationship between soluble Aβ levels and ApoE concentrations in either treatment group (data not shown). This finding suggests that alterations in ApoE levels are not responsible for the elevation in soluble Aβ levels that are observed in the brains of ABX-treated male APP_SWE_/PS1_ΔE9_ mice.

In parallel studies, we assessed Aβ burden and Aβ levels in female APP_SWE_/PS1_ΔE9_ mice treated with vehicle or ABX. Female mice were sacrificed at 5 months of age as it is well established that Aβ deposition occurs earlier in female APP_SWE_/PS1_ΔE9_ mice as compared to males. In both immunocytochemical assessments of Aβ burden or biochemical analyses of Aβ levels, we failed to observe any differences in these parameters between ABX-treated versus vehicle-treated control mice (*n* = 8–10, Supp. Fig. 9A–I).

### Reactive gliosis surrounding Aβ plaques is reduced in male ABX-treated APP_SWE_/PS1_ΔE9_ mice

**Figure 4:**
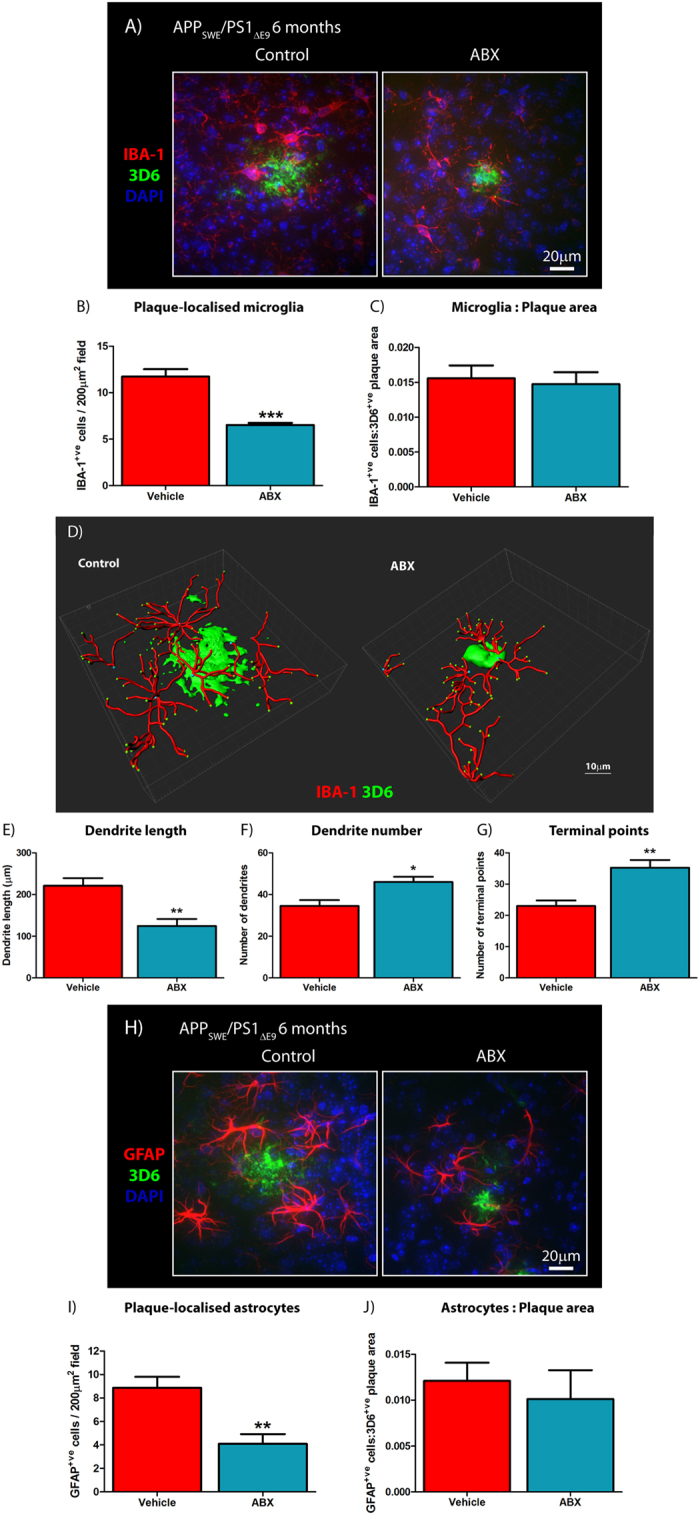
Plaque-localized gliosis is influenced by ABX treatment in male APP_SWE_/PS1_ΔE9_ mice. (**A**) Representative x60 magnification z-stack maximum projection images of IBA-1^+ve^ Aβ plaque-localized microglia, co-stained with DAPI, in vehicle control and ABX-treated male 6 month old APP_SWE_/PS1_ΔE9_ mice. (**B**) Quantification of plaque-localized IBA-1^+ve^ microglial number in vehicle control and ABX-treated male 6 month old APP_SWE_/PS1_ΔE9_ mice (*n* = 8, ***p < 0.001, unpaired two-tailed Student’s *t*-test). (**C**) Plaque-localized IBA^+ve^ microglial number expressed relative to 3D6^+ve^ Aβ plaque area in vehicle control and ABX-treated male 6 month old APP_SWE_/PS1_ΔE9_ mice (*n* = 8). (**D**) Representative 3D IMARIS-based reconstructions of x60 magnification z-stack images (0.8 μm apart) depicting plaque-localized IBA-1^+ve^ microglial morphology in in vehicle control and ABX-treated male 6 month old APP_SWE_/PS1_ΔE9_ mice. Microglial branching, terminal points and Aβ plaque surfaces can be seen. The (**E**) length and (**F**) number of microglial dendritic branches alongside (**G**) projection terminal points were quantified (*n* = 4, *p = 0.0237, **p < 0.01, unpaired two-tailed Student’s *t*-test). (**H**) Representative x60 magnification z-stack maximum projection images of GFAP^+ve^ Aβ plaque-localized astrocytes, co-stained with DAPI, in vehicle control and ABX-treated male 6 month old APP_SWE_/PS1_ΔE9_ mice. (**I**) Quantification of plaque-localized GFAP^+ve^ astrocyte number in vehicle control and ABX-treated male 6 month old APP_SWE_/PS1_ΔE9_ mice (*n* = 8, **p = 0.001, unpaired two-tailed Student’s *t*-test). (**J**) Plaque-localized GFAP^+ve^ astrocyte number expressed relative to 3D6^+ve^ Aβ plaque area in vehicle control and ABX-treated male 6 month old APP_SWE_/PS1_ΔE9_ mice (*n* = 8). Data are displayed as mean ± SEM. See statistical table 1 for additional information.

To assess potential alterations in neuro-inflammatory state and reactive gliosis, we analyzed Aβ plaque-localized microglial populations by immunohistochemistry using an antibody to ionized calcium binding adaptor molecule 1 (Iba1), a microglia/macrophage-specific calcium-binding protein. Analysis of high power x60 magnification z-stack images revealed a significant 1.79-fold decrease in plaque-localized Iba-1 positive microglial cells in male ABX-treated APP_SWE_/PS1_ΔE9_ mice compared with vehicle controls (11.73 ± 0.79 vs. 6.53 ± 0.21 cells/200 μm^2^ field, *n* = 8, p = 0.001, Fig. 4A,B). However, this difference was not apparent when the number of Iba1-positive cells was expressed relative to Aβ plaque area stained using mAb 3D6 (*n* = 8, Fig. 4C).

As morphology and reactivity of microglia is regulated by gut microbial communities^28^, we generated 3D-reconstructions of these plaque-localized Iba1-positive microglia identified by immunohistochemistry. 3D-IMARIS-based reconstructions from z-stack immunohistochemistry images revealed altered microglial morphology in male ABX-treated APP_SWE_/PS1_ΔE9_ mice compared to vehicle controls (Fig. 4D). Quantification of these re-constructions revealed a 1.78-fold decrease in dendrite length (221.2 ± 18.17 vs. 124.5 ± 16.81 μm, *n* = 4, p-0.008, Fig. 4E), a 1.33-fold increase in dendrite number (34.5 ± 2.84 vs. 46.0 ± 2.55, *n* = 4, p = 0.0237, Fig. 4F) and a 1.53-fold increase in terminal end-points (23.0 ± 1.78 vs. 35.25 ± 2.46, *n* = 4, p = 0.007, Fig. 4G) of plaque-localized microglia in male ABX-treated APP_SWE_/PS1_ΔE9_ mice compared with vehicle-treated control animals. Collectively, these findings identify reduced numbers and altered morphology of Aβ plaque-localized microglia in male ABX-treated APP_SWE_/PS1_ΔE9_ mice.

In addition to microglia driving neuro-inflammatory responses in the CNS, astrocytes are also important contributors. Hence, we performed immunocytochemistry with an antibody specific for glial fibrillary acidic protein (GFAP) that is highly enriched in astrocytes. Analysis of high power x60 magnification z-stack images revealed a significant 2.17-fold decrease in plaque-localized GFAP-positive astrocytes in male ABX-treated APP_SWE_/PS1_ΔE9_ mice compared with vehicle controls (8.87 ± 0.94 vs. 4.09 ± 0.83 cells/200 μm^2^ field, *n* = 8, p = 0.001, Fig. 4H,I). Similar to the microglial analysis, this difference was not apparent when the number of GFAP-positive cells was expressed relative to Aβ plaque area (*n* = 8, Fig. 3J). These findings confirm that similar to microglia, astrocyte reactivity surrounding Aβ deposits is reduced in brains of male ABX-treated APP_SWE_/PS1_ΔE9_ mice.

## Discussion

It has become abundantly clear that the gut microbiota play a complex role in regulating innate immunity and brain function. The present study was designed to test the hypothesis that the composition of the intestinal microbiome might play a role in modulating neuro-inflammation in a manner that could influence amyloid plaque deposition in a mouse model of Aβ amyloidosis. We now offer several important insights. First, we demonstrate that long-term ABX treatment induces a distinct perturbation in gut microbial diversity and alters peripherally circulating cytokine/chemokine composition. Second, we observe a striking reduction in amyloid plaque deposition and elevated levels of soluble Aβ in male APP_SWE_/PS1_ΔE9_ mice. Finally, ABX-induced perturbations in gut microbial diversity also influenced neuro-inflammatory responses by conferring reduced plaque-localized gliosis and altered microglial morphology.

Our demonstration that ABX-treatment of APP_SWE_/PS1_ΔE9_ mice leads to alterations in several circulating inflammatory chemokines and cytokines in the blood is notable. Amongst these molecules, we were particularly intrigued by the observation that serum levels of CCL11 are elevated in the serum of ABX-treated mice. CCL11 has been linked to age-associated deficits in hippocampal neurogenesis^38^, and the chemokine gene cluster containing CCL11 has recently been implicated as a risk factor for late-onset AD^39^. CCL11 is known to cross the blood brain barrier^40^ and we speculate would lead to microglial activation and subsequent phagocytosis of Aβ deposits. Consistent with this model, our 3D reconstructions reveal that although the numbers of microglia surrounding plaques are diminished in brains of ABX-treated mice, these cells appear to be highly ramified and thus, in an activated state. It is equally plausible that the microglia have been activated throughout the course of ABX treatment and that these cells are continually clearing any deposited Aβ or perhaps, oligomeric forms of the peptide. Thus, temporal elevations in peripheral cytokine and chemokine levels at this early time-point may facilitate a beneficial neuro-inflammation state that clears amyloid, but enhanced cytokine load combined with the deleterious effects of aging may worsen disease pathology. In support of this concept is the demonstration that genetic deletion of TLR4, required for efficient microglial detection of Aβ, is sufficient in dampening early-stage neuro-inflammatory responses leading to enhanced amyloidosis and impaired cognitive function in APP_SWE_/PS1_ΔE9_ mice^41^. Additionally, genetic ablation of anti-inflammatory IL-10 leads to a re-balancing of microglial-based innate immunity and attenuates plaque pathology in APP_SWE_/PS1_ΔE9_ mice^14^.

We are fully cognizant of the fact that the findings reported herein are purely correlative and do not elucidate precise mechanism(s). Nevertheless, our findings indicate that a chronic, staged ABX regimen is able to establish a stable, but altered state of the gut microbiota that is associated with changes in glial and immune functions capable of mitigating Aβ amyloidosis. In essence, the ABX regimen reveals that the microbiome can be altered in ways to achieve states of host-microbe interaction that affect immune responses systemically and, in this AD model, prevents the natural progression of disease. It will be critical to extend these observations to determine how this happens. Was it through the loss of microbiome-derived pro-inflammatory factors, or through the gain of disease-preventing factors from a new steady state community development of gut microbiota? The answers to these questions, while out of the scope of this study, will lead to the development of practical microbiome-based interventions for human subjects at risk or in the early stages of AD. Moreover, the enigmatic finding that the levels of soluble Aβ were elevated in the brains of ABX-treated male APP_SWE_/PS1_ΔE9_ mice that displayed reduced insoluble Aβ levels and plaque burden suggests future microbiome-based therapies may afford an opportunity to intervene in specific pathophysiological events and change the natural history of disease. Finally, the nature of the metabolites produced by different microbial communities in our mouse model and the potential impact of these metabolites amyloid deposition remain to be determined. Future efforts will focus on clarification of these important issues with the premise that these studies will offer new insights into the regulation of Aβ deposition by innate immune pathways influenced by the gut microbiome and ultimately, the development of novel therapeutic modalities for AD.

## Methods

### Animals and antibiotic treatment regime

All murine experimental procedures were approved by the Institutional Animal Care and Use Committee (IACUC) at the University of Chicago and performed in accordance with approved Animal Care and Use Protocols (ACUPs). Male and female APP_SWE_/PS1_ΔE9_ transgenic mice on a hybrid C57BL/6-C3-hej genetic background (B6C3-Tg(APPswe,PSEN1dE9)85Dbo/Mmjax, JAX ID: 004462, www.jax.org/strain/004462) were determined as specific pathogen-free, housed in sterile micro-isolator cages and fed *ad-libitum* on standard chow. Mice were gavaged with combinatorial antibiotics (ABX, gentamicin (1 mg/ml), vancomycin (0.5 mg/ml), metronidazole (2 mg/ml), neomycin (0.5 mg/ml), ampicillin (1 mg/ml), kanamycin (3 mg/ml), colistin (6000 U/ml) and cefaperazone (1 mg/ml), Sigma) from postnatal day 14 until day 21 and were then supplemented with ABX-containing drinking water (1/50^th^ gavage concentration) for the duration of lifespan^25^,^42^. Efficacy of ABX treatment was tracked by monitoring bacterial growth from fecal homogenates on LB agar plates and in LB media throughout the treatment course (Supp. Fig. 1A,B). Enlargement of the cecum, which is considered indicative of effective microbial suppression, in ABX-treated animals was also confirmed (Supp. Fig. 1C). Mice assigned to vehicle control groups were subjected to the same treatment protocol with autoclaved water only. Male mice were killed at 6 months of age and females were killed at 5 months of age for subsequent experimentation.

### Cardiac perfusion of mice and tissue harvesting

Mice were deeply anaesthetized using inhaled isoflurane, USP (Piramal Healthcare). Mice were cardiac perfused with ice-cold heparinized PBS (1 U/ml, Sigma). Brains were then excised and separated for use in immunohistochemistry or for RNA/protein biochemical analysis. Isolation of the cortex for RNA/protein biochemistry was performed using a modified dissection technique^43^. Hemispheres were placed on an ice cold glass dissection plate and orientated in a sagittal plane. The cerebellum was removed, and the striatum, thalamus, midbrain and brain stem remnants were identified. These structures were then removed using sterilized blunt spatulas, exposing the hippocampal complex and interior wall of the cortex. Combined cortical and hippocampal tissue was snap frozen in liquid nitrogen and stored at −80 °C until required.

Cecal contents and whole spleens were also obtained and were snap frozen in liquid nitrogen followed by storage at −80 °C until required.

### Cecal and Fecal DNA extraction

Extraction of bacterial DNA from cecal and fecal contents was performed as previously described^44^ and conforms to standardized earth microbiome project protocols (www.earthmicrobiome.org/emp-standard-protocols/). Briefly, tissues were dissolved in extraction buffer (50 mg tissue/ml buffer, 50 mM Tris (pH 7.4), 100 mM EDTA (pH 8.0), 400 mM NaCl, 0.5% w/v SDS) containing proteinase K (0.4 mg/ml). After addition of 0.1-mm diameter glass beads (500 ul/ml buffer, BioSpec Products), microbial cells were lysed using a Mini-Beadbeater-8K Cell Disrupter (BioSpec Products) and overnight water bath incubation (55 °C). Total DNA was then extracted using Phenol:Chloroform:IAA (25:24:1 v/v, pH 8.0, Ambion) according to manufacturer’s protocols. DNA yield was quantified and quality ascertained by a combination of Nanodrop Lite (Thermo Fisher) and Qubit® fluorometer (Invitrogen) assessment.

### 16s rRNA gene quantitative polymerase chain reaction (Q-PCR)

16s rRNA gene copy number was quantified from DNA isolated from fecal and cecal contents using QPCR. 2–5 ng/ul DNA was added to iQ-SYBR green PCR supermix (BioRad) with 518F (5′-TCC-TAC-GGG-AGG-CAG-CAG-T-3′) and 338R (5′-GGA-CTA-CCA-GGG-TAT-CTA-ATC-CTG-TT-3′) primers (2.5 μM) and Q-PCR was performed on a Lightcycler® 480 system (Roche) under the following parameters: 95 °C for 5 minutes, (95 °C for 10 seconds, 64 °C for 45 seconds, 72 °C for 45 seconds) x35 cycles, 40 °C for 30 seconds.

16s rRNA gene copy number was determined by reference of Cp values to a standard curve of the pCR4-TOPO plasmid inclusive of the 16s rRNA gene amplicon. Copy number was then expressed relative to the precise DNA concentration added per reaction as determined by earlier Qubit® fluorometer (Invitrogen) assessment. All reactions were conducted in triplicate with appropriate negative controls.

### 16s rRNA gene Illumina® MiSeq sequencing

Deep sequencing of the 16s rRNA gene from cecal and fecal contents was performed as previously described^45^. The V4-V5 region of the 16s rRNA gene was amplified using standard PCR methods (www.earthmicrobiome.org/emp-standard-protocols) from total DNA. Illumina® MiSeq gene sequencing was then performed the Institute for Genomics and Systems Biology’s Next Generation Sequencing Core at Argonne National Laboratory. Sequences were trimmed and classified using QIIME version 1.7^46^. Operational taxonomic units (OTUs) were picked at 97% sequence identity using open-reference OTU picking against the Greengenes database version 12_10^47^. These quality-controlled sequences were then aligned using PyNAST^48^, taxonomy was assigned using the RDP classifier^49^ and a phylogenetic tree was constructed using FastTree version 2.0^50^. Shannon indices, indicative of α-diversity were then calculated from rarefaction plots. Un-weighted and weighted UniFrac distances were then computed to produce β-diversity dissimilarity matrices^51^.

### Immunohistochemistry

For immunohistochemistry, hemispheres not used for biochemical analysis were post-fixed in 4% w/v paraformaldehyde, 0.1% v/v glutaraldehyde for 72 hrs and subsequently submerged in 30% w/v sucrose for cryoprotective purposes. Hemispheres were then placed in OCT freezing medium and mounted on a Leica SM2000R freezing microtome stage (Leica Biosystems). Hemispheres were then serially sectioned at 40 μm thickness in a coronal plane through the hippocampal complex from −1.5 to −3.0 bregma cordinates (Paxinos G, Mouse brain atlas) and sections were stored in cryoprotective solution (25% v/v glycerin and 30% v/v ethylene glycol in PBS, Fischer Scientific) at −20 °C until required. Every 6^th^ section (spaced 240 μm apart) was used for staining and subsequent quantification.

Sections were heated to 95 °C in SSC buffer (0.15 M Sodium Chloride, 0.15 M Sodium Citrate, pH7.0) for 20 min to aid in antigen presentation and then blocked in 5% v/v donkey serum in TBS-T (0.25% v/v Triton X-100, Sigma) for 2 hrs at room temperature. Sections were then incubated with unconjugated primary antibodies for 24 hrs at 4 °C. After washing with TBS-T, sections were then incubated with fluorophore-conjugated secondary antibodies for 3 hrs at room temperature whilst protected from light. After sequential washing with TBS-T, TBS and DEPC-treated H_2_O, sections were mounted on Superfrost® plus slides (Fisher Scientific) using VectaShield® mounting medium containing DAPI (Vecta Laboratories). The primary antibodies used were a mouse monoclonal anti-Aβ (3D6, 190 pg/ml final concentration, in-house purified), a rabbit polyclonal anti-ionized calcium-binding adapter molecule 1 (IBA-1, 1:500, 019-19741, Wako) and a rabbit monoclonal anti-glial fibrillary acidic protein (GFAP, 1:2000, clone D1F4Q, 12389S, Cell Signaling). The fluorophore-conjugated secondary antibodies used were a donkey anti-mouse IgG Alexa Fluor® 488 conjugate (1:1000, A21202, Life Technologies) and a donkey anti-rabbit IgG Alexa Fluor® 594 (1:1000, A21207, Life Technologies). Whole sections were then imaged at x20 magnification using the Pannoramic SCAN BF plus FL Optimum slide scanner (PerkinElmer/3DHistech) or individual plaques at x60 magnification using the Olympus IX2-series spinning DSU confocal inverted microscope (Olympus).

### Amyloid plaque quantification and cell counting

To quantify plaque burden, brain sections prepared as above, were analyzed as 8-Bit images using ImageJ software (NIH). For each section intensity thresholds were set to eliminate background fluorescence and plaque staining was analyzed by particle quantification. This value was then expressed relative to the calculated area of the combined cortical and hippocampal region of each individual section. This value was then averaged from 4 sections per mouse to calculate a plaque burden percentage for each mouse throughout the study. Individual plaque size was calculated using a similar method but also directly from x60 z-stack images. Plaque-localized microglial and astrocyte cell numbers were counted using a combination of ImageJ and Stereo Investigator (MBF Bioscience) software packages. IBA-1^+ve^ and GFAP^+ve^ positive somas co-localised with DAPI and within the 200 μm vicinity of 3D6^+ve^ Aβ plaque fluorescence were identified as plaque-localised microglia and astrocytes respectively.

### Three-dimensional reconstruction of plaque-localised microglia

Sections co-labelled with anti-Aβ and anti-IBA-1 antibodies and counter-stained with DAPI for nuclei were prepared as above. Z-stack images of individual plaques from 40 μm thick sections were obtained under x60 magnification and water immersion with 0.8 μm step increments in the *z* plane. These z-stack images were then recorded and analysed using IMARIS software (Bitplane). The surface tool was used to establish the realm of the Aβ plaque and then the filament tool was used to map microglial cell bodies and dendrite-like processes. 4 plaque-localized microglia were analysed per mouse.

### MSD MesoScale Aβ ELISA

For quantification of soluble and insoluble Aβ levels, brain tissues were ground on LN_2_ and were homogenized in Tris-buffered saline (TBS) containing protease inhibitors (Roche) via sonication and polytron processing. After centrifugation (100,000 × g, 60 min, 4 °C) the supernatant was collected to detect TBS soluble Aβ levels. The remaining pellets were further homogenized in 70% v/v formic acid in TBS (TFA, Sigma) via polytron processing, centrifuged (100,000 × g, 60 min, 4 °C), and the resulting supernatant was collected to detect TBS-insoluble Aβ levels. TFA samples were neutralized in 1 M Tris buffer (20× volume) prior to Meso Scale analysis.

Aβ levels were quantified using Meso Scale Aβ38/40/42-triplex kits (V-PLEX Aβ Peptide Panel 1 (4G8) Kit, K15199E-1, Meso Scale Diagnostics), as previously described^52^,^53^,^54^. Assays were run in 96-well plate format with all standards and sample run in duplicate. Electro-chemiluminescence signals were captured by the MESO QuickPlex SQ 120 system (Meso Scale Diagnostics). Sample Aβ38, Aβ40 and Aβ42 levels were normalized to the Aβ standard curve and concentrations are expressed relative to sample total protein concentrations as determined by BCA assay (Thermo Fisher). Whilst, Aβ40 and Aβ42 proteins were robustly detected in samples assayed, Aβ38 failed to be detected.

### Statistical analysis

GraphPad Prism software (version 6.0, www.graphpad.com/scientific-software/prism/) was used for all unpaired two-tailed Student’s *t*-tests and Pearson’s multi-variate linear regression analysis. For all statistical tests a two-tailed α value of 0.05 was utilised. All numerical data is presented as X/Y scatter, mean alone, or, mean ± standard error of the mean (SEM). Power values for each test where calculated post-hoc using G*Power (version 3.1, gpower.hhu.de/), based upon the effect size, group number and sample size. Exact p-values were calculated for all Student’s *t*-tests. A p-value < 0.05 was considered statistically significant. All use of statistics is detailed in supplementary statistics Table 1.

### Data availability

MiSeq 16s rRNA V4 amplicon sequencing data has been uploaded to the European Bioinformatics Institute (EBI) database using Qiita (project ID: 10516).

## Additional Information

**How to cite this article**: Minter, M. R. *et al.* Antibiotic-induced perturbations in gut microbial diversity influences neuro-inflammation and amyloidosis in a murine model of Alzheimer’s disease. *Sci. Rep.* **6**, 30028; doi: 10.1038/srep30028 (2016).

## Supplementary Material

Supplementary Information

## References

[b1] Arends Y. M., Duyckaerts C., Rozemuller J. M., Eikelenboom P. & Hauw J. J. Microglia, amyloid and dementia in alzheimer disease. A correlative study. Neurobiology of aging 21, 39–47 (2000). 10794847 10.1016/s0197-4580(00)00094-4

[b2] Beach T. G., Walker R. & McGeer E. G. Patterns of gliosis in Alzheimer’s disease and aging cerebrum. Glia 2, 420–436, doi: 10.1002/glia.440020605 (1989). 2531723

[b3] Delacourte A. General and dramatic glial reaction in Alzheimer brains. Neurology 40, 33–37 (1990). 2296379 10.1212/wnl.40.1.33

[b4] Cagnin A. *et al.* *In-vivo* measurement of activated microglia in dementia. Lancet 358, 461–467, doi: 10.1016/S0140-6736(01)05625-2 (2001). 11513911

[b5] Yasuno F. *et al.* Increased binding of peripheral benzodiazepine receptor in mild cognitive impairment-dementia converters measured by positron emission tomography with [(1)(1)C]DAA1106. Psychiatry research 203, 67–74, doi: 10.1016/j.pscychresns.2011.08.013 (2012). 22892349

[b6] Griciuc A. *et al.* Alzheimer’s disease risk gene CD33 inhibits microglial uptake of amyloid beta. Neuron 78, 631–643, doi: 10.1016/j.neuron.2013.04.014 (2013). 23623698 PMC3706457

[b7] Guerreiro R. *et al.* TREM2 variants in Alzheimer’s disease. The New England journal of medicine 368, 117–127, doi: 10.1056/NEJMoa1211851 (2013). 23150934 PMC3631573

[b8] Jonsson T. *et al.* Variant of TREM2 associated with the risk of Alzheimer’s disease. The New England journal of medicine 368, 107–116, doi: 10.1056/NEJMoa1211103 (2013). 23150908 PMC3677583

[b9] Lambert J. C. *et al.* Genome-wide association study identifies variants at CLU and CR1 associated with Alzheimer’s disease. Nature genetics 41, 1094–1099, doi: 10.1038/ng.439 (2009). 19734903

[b10] Naj A. C. *et al.* Common variants at MS4A4/MS4A6E, CD2AP, CD33 and EPHA1 are associated with late-onset Alzheimer’s disease. Nature genetics 43, 436–441, doi: 10.1038/ng.801 (2011). 21460841 PMC3090745

[b11] Naj A. C. *et al.* Effects of multiple genetic loci on age at onset in late-onset Alzheimer disease: a genome-wide association study. JAMA neurology 71, 1394–1404, doi: 10.1001/jamaneurol.2014.1491 (2014). 25199842 PMC4314944

[b12] Minter M. R., Taylor J. M. & Crack P. J. The contribution of neuroinflammation to amyloid toxicity in Alzheimer’s disease. Journal of neurochemistry 136, 457–474, doi: 10.1111/jnc.13411 (2016). 26509334

[b13] Chakrabarty P. *et al.* IL-10 alters immunoproteostasis in APP mice, increasing plaque burden and worsening cognitive behavior. Neuron 85, 519–533, doi: 10.1016/j.neuron.2014.11.020 (2015). 25619653 PMC4320003

[b14] Guillot-Sestier M. V. *et al.* Il10 deficiency rebalances innate immunity to mitigate Alzheimer-like pathology. Neuron 85, 534–548, doi: 10.1016/j.neuron.2014.12.068 (2015). 25619654 PMC4352138

[b15] He P. *et al.* Deletion of tumor necrosis factor death receptor inhibits amyloid beta generation and prevents learning and memory deficits in Alzheimer’s mice. The Journal of cell biology 178, 829–841, doi: 10.1083/jcb.200705042 (2007). 17724122 PMC2064547

[b16] Heneka M. T. *et al.* NLRP3 is activated in Alzheimer’s disease and contributes to pathology in APP/PS1 mice. Nature 493, 674–678, doi: 10.1038/nature11729 (2013). 23254930 PMC3812809

[b17] Vom Berg J. *et al.* Inhibition of IL-12/IL-23 signaling reduces Alzheimer’s disease-like pathology and cognitive decline. Nature medicine 18, 1812–1819, doi: 10.1038/nm.2965 (2012). 23178247

[b18] Baruch K. *et al.* Aging. Aging-induced type I interferon response at the choroid plexus negatively affects brain function. Science 346, 89–93, doi: 10.1126/science.1252945 (2014). 25147279 PMC4869326

[b19] Taylor J. M. *et al.* Type-1 interferon signaling mediates neuro-inflammatory events in models of Alzheimer’s disease. Neurobiology of aging 35, 1012–1023, doi: 10.1016/j.neurobiolaging.2013.10.089 (2014). 24262201

[b20] Baruch K. *et al.* Breaking immune tolerance by targeting Foxp3(+) regulatory T cells mitigates Alzheimer’s disease pathology. Nature communications 6, 7967, doi: 10.1038/ncomms8967 (2015). PMC4557123 26284939

[b21] Gyoneva S. *et al.* Systemic inflammation regulates microglial responses to tissue damage *in vivo*. Glia 62, 1345–1360, doi: 10.1002/glia.22686 (2014). 24807189 PMC4408916

[b22] Sherwin E., Rea K., Dinan T. G. & Cryan J. F. A gut (microbiome) feeling about the brain. Current opinion in gastroenterology 32, 96–102, doi: 10.1097/MOG.0000000000000244 (2016). 26760398

[b23] Kim H. R. *et al.* Delivery modes and neonatal EEG: spatial pattern analysis. Early human development 75, 35–53 (2003). 14652158 10.1016/j.earlhumdev.2003.09.004

[b24] Juarez I., Gratton A. & Flores G. Ontogeny of altered dendritic morphology in the rat prefrontal cortex, hippocampus, and nucleus accumbens following Cesarean delivery and birth anoxia. The Journal of comparative neurology 507, 1734–1747, doi: 10.1002/cne.21651 (2008). 18253967

[b25] Bashir M. E., Louie S., Shi H. N. & Nagler-Anderson C. Toll-like receptor 4 signaling by intestinal microbes influences susceptibility to food allergy. Journal of immunology 172, 6978–6987 (2004). 10.4049/jimmunol.172.11.6978 15153518

[b26] Hormann N. *et al.* Gut microbial colonization orchestrates TLR2 expression, signaling and epithelial proliferation in the small intestinal mucosa. PloS one 9, e113080, doi: 10.1371/journal.pone.0113080 (2014). 25396415 PMC4232598

[b27] Wang Y. *et al.* An intestinal commensal symbiosis factor controls neuroinflammation via TLR2-mediated CD39 signalling. Nature communications 5, 4432, doi: 10.1038/ncomms5432 (2014). PMC4118494 25043484

[b28] Erny D. *et al.* Host microbiota constantly control maturation and function of microglia in the CNS. Nature neuroscience 18, 965–977, doi: 10.1038/nn.4030 (2015). 26030851 PMC5528863

[b29] Jankowsky J. L. *et al.* Mutant presenilins specifically elevate the levels of the 42 residue beta-amyloid peptide *in vivo*: evidence for augmentation of a 42-specific gamma secretase. Human molecular genetics 13, 159–170, doi: 10.1093/hmg/ddh019 (2004). 14645205

[b30] Jankowsky J. L. *et al.* Co-expression of multiple transgenes in mouse CNS: a comparison of strategies. Biomolecular engineering 17, 157–165 (2001). 11337275 10.1016/s1389-0344(01)00067-3

[b31] Lathrop S. K. *et al.* Peripheral education of the immune system by colonic commensal microbiota. Nature 478, 250–254, doi: 10.1038/nature10434 (2011). 21937990 PMC3192908

[b32] Sun J. *et al.* Pancreatic beta-Cells Limit Autoimmune Diabetes via an Immunoregulatory Antimicrobial Peptide Expressed under the Influence of the Gut Microbiota. Immunity 43, 304–317, doi: 10.1016/j.immuni.2015.07.013 (2015). 26253786

[b33] Zhang Z. *et al.* Peripheral Lymphoid Volume Expansion and Maintenance Are Controlled by Gut Microbiota via RALDH(+) Dendritic Cells. Immunity 44, 330–342, doi: 10.1016/j.immuni.2016.01.004 (2016). 26885858 PMC4757854

[b34] Johnson-Wood K. *et al.* Amyloid precursor protein processing and A beta42 deposition in a transgenic mouse model of Alzheimer disease. Proceedings of the National Academy of Sciences of the United States of America 94, 1550–1555 (1997). 9037091 10.1073/pnas.94.4.1550 PMC19829

[b35] Garai K., Verghese P. B., Baban B., Holtzman D. M. & Frieden C. The binding of apolipoprotein E to oligomers and fibrils of amyloid-beta alters the kinetics of amyloid aggregation. Biochemistry 53, 6323–6331, doi: 10.1021/bi5008172 (2014). 25207746 PMC4196732

[b36] Liao F. *et al.* Anti-ApoE antibody given after plaque onset decreases Abeta accumulation and improves brain function in a mouse model of Abeta amyloidosis. The Journal of neuroscience: the official journal of the Society for Neuroscience 34, 7281–7292, doi: 10.1523/JNEUROSCI.0646-14.2014 (2014). 24849360 PMC4028501

[b37] Liao F. *et al.* Murine versus human apolipoprotein E4: differential facilitation of and co-localization in cerebral amyloid angiopathy and amyloid plaques in APP transgenic mouse models. Acta neuropathologica communications 3, 70, doi: 10.1186/s40478-015-0250-y (2015). 26556230 PMC4641345

[b38] Villeda S. A. *et al.* The ageing systemic milieu negatively regulates neurogenesis and cognitive function. Nature 477, 90–94, doi: 10.1038/nature10357 (2011). 21886162 PMC3170097

[b39] Lalli M. A. *et al.* Whole-genome sequencing suggests a chemokine gene cluster that modifies age at onset in familial Alzheimer’s disease. Molecular psychiatry 20, 1294–1300, doi: 10.1038/mp.2015.131 (2015). 26324103 PMC4759097

[b40] Erickson M. A., Morofuji Y., Owen J. B. & Banks W. A. Rapid transport of CCL11 across the blood-brain barrier: regional variation and importance of blood cells. The Journal of pharmacology and experimental therapeutics 349, 497–507, doi: 10.1124/jpet.114.213074 (2014). 24706984 PMC4019322

[b41] Song M. *et al.* TLR4 mutation reduces microglial activation, increases Abeta deposits and exacerbates cognitive deficits in a mouse model of Alzheimer’s disease. Journal of neuroinflammation 8, 92, doi: 10.1186/1742-2094-8-92 (2011). 21827663 PMC3169468

[b42] Stefka A. T. *et al.* Commensal bacteria protect against food allergen sensitization. Proceedings of the National Academy of Sciences of the United States of America 111, 13145–13150, doi: 10.1073/pnas.1412008111 (2014). 25157157 PMC4246970

[b43] Hagihara H., Toyama K., Yamasaki N. & Miyakawa T. Dissection of hippocampal dentate gyrus from adult mouse. J Vis Exp, doi: 10.3791/1543 (2009). PMC3142893 19920804

[b44] Wang Y. *et al.* 16S rRNA gene-based analysis of fecal microbiota from preterm infants with and without necrotizing enterocolitis. The ISME journal 3, 944–954, doi: 10.1038/ismej.2009.37 (2009). 19369970 PMC2713796

[b45] Leone V. *et al.* Effects of diurnal variation of gut microbes and high-fat feeding on host circadian clock function and metabolism. Cell host & microbe 17, 681–689, doi: 10.1016/j.chom.2015.03.006 (2015). 25891358 PMC4433408

[b46] Caporaso J. G. *et al.* QIIME allows analysis of high-throughput community sequencing data. Nature methods 7, 335–336, doi: 10.1038/nmeth.f.303 (2010). 20383131 PMC3156573

[b47] McDonald D. *et al.* An improved Greengenes taxonomy with explicit ranks for ecological and evolutionary analyses of bacteria and archaea. The ISME journal 6, 610–618, doi: 10.1038/ismej.2011.139 (2012). 22134646 PMC3280142

[b48] Caporaso J. G. *et al.* PyNAST: a flexible tool for aligning sequences to a template alignment. Bioinformatics 26, 266–267, doi: 10.1093/bioinformatics/btp636 (2010). 19914921 PMC2804299

[b49] Wang Q., Garrity G. M., Tiedje J. M. & Cole J. R. Naive Bayesian classifier for rapid assignment of rRNA sequences into the new bacterial taxonomy. Applied and environmental microbiology 73, 5261–5267, doi: 10.1128/AEM.00062-07 (2007). 17586664 PMC1950982

[b50] Price M. N., Dehal P. S. & Arkin A. P. FastTree 2–approximately maximum-likelihood trees for large alignments. PloS one 5, e9490, doi: 10.1371/journal.pone.0009490 (2010). 20224823 PMC2835736

[b51] Lozupone C. & Knight R. UniFrac: a new phylogenetic method for comparing microbial communities. Applied and environmental microbiology 71, 8228–8235, doi: 10.1128/AEM.71.12.8228-8235.2005 (2005). 16332807 PMC1317376

[b52] Wagner S. L. *et al.* Soluble gamma-secretase modulators selectively inhibit the production of Abeta42 and augment the production of multiple carboxy-truncated Abeta species. Biochemistry, doi: 10.1021/bi401537v (2014). PMC3929337 24401146

[b53] Choi S. H. *et al.* A three-dimensional human neural cell culture model of Alzheimer’s disease. Nature, doi: 10.1038/nature13800 (2014). PMC4366007 25307057

[b54] Zhang X. *et al.* Near-infrared fluorescence molecular imaging of amyloid beta species and monitoring therapy in animal models of Alzheimer’s disease. Proceedings of the National Academy of Sciences of the United States of America 112, 9734–9739, doi: 10.1073/pnas.1505420112 (2015). 26199414 PMC4534214

