# Supplementary material for: Antibiotic-induced perturbations in gut microbial diversity influences neuro-inflammation and amyloidosis in a murine model of Alzheimer’s disease
Source: Sci Rep. 2016 Jul 21;6:30028. doi: 10.1038/srep30028 (PMC4956742; doi:10.1038/srep30028)
Supplement: Supplementary Information [file srep30028-s1.doc]

**Supplementary Material**

**Antibiotic-induced perturbations in gut microbial diversity influences neuro-inflammation and amyloidosis in a murine model of Alzheimer’s disease.**

Myles R. Minter^1,5^, Can Zhang^2^, Vanessa Leone^3,5^, Daina L. Ringus^3,5^, Xiaoqiong Zhang^1^, Paul Oyler-Castrillo^1^, Mark W. Musch^3,5^, Fan Liao^4^, Joseph F. Ward^2^, David M Holtzman^4^, Eugene B. Chang^3,5^, Rudolph E. Tanzi^2^ and Sangram S. Sisodia^1,5^*.

^1^Department of Neurobiology, The University of Chicago, Chicago, IL, 60637, USA

^2^Department of Neurology, Genetics and Aging Research Unit, MassGeneral Institute for Neurodegenerative Diseases, Massachusetts General Hospital, Charlestown, MA 02114, USA

^3^Department of Medicine, The University of Chicago, Chicago, IL, 60637, USA

^4^Department of Neurology, Hope Center for Neurological Disorders, Charles F. and Joanne Knight Alzheimer's Disease Research Center, Washington University School of Medicine, St. Louis, MO, 63110, USA

^5^The Microbiome Center, The University of Chicago, Chicago, IL, 60637, USA

*Correspondence: ssisodia@bsd.uchicago.edu

**Supplementary methods**

**Bacterial culture from fecal homogenates**

Fresh fecal pellets were obtained from animals and homgenised in 1ml LB medium (Fisher Scientific) via repetitive pipetting under aseptic conditions. Fecal homogenates were then spread on LB agar plates (Invitrogen), sealed with laboratory parafilm and incubated overnight at 37ᵒC. Fecal homogenates were also grown in 5ml LB medium overnight at 37ᵒC with shaking (225rpm) and optical density (O.D.) of cultures was measured at 600nm using an ELx808 multi-channel absorbance reader (BioTek) to track bacterial growth rates. To test antibiotic resistance in APP_SWE_/PS1_ΔE9_ mice treated with ABX, fecal homogenates from 6 month old mice were obtained and grown in LB medium containing ABX (1/50^th^ gavage concentration, see primary methods) in aforementioned conditions. O.D._600nm_ values were then determined as above.

**16s rRNA gene PCR and Terminal restriction fragment length polymorphism (T-RFLP) analysis**

T-RFLP analysis of the 16s rRNA gene product from cecal and fecal bacterial DNA was performed as previously described[^1^](#_ENREF_1). The 16s rRNA gene was amplified using primers (0.25µM) 8F (5’-AGA-GTT-TGA-TCC-TGG-CTC-AGT-3’), labelled with 6’-FAM at the 5’ end, and 1492R (5’-GGT-TAC-CTT-GTT-ACG-ACT-T-3’) in conjunction with the Takara® Ex-Taq polymerase system (Takara) under the following thermal cycler parameters:

| Step | Temperature | Time | Repeats |
| --- | --- | --- | --- |
| 1 | 94ᵒC | 10min | 1 |
| 2 | 94ᵒC | 30sec | 30 |
| 3 | 55ᵒC | 30sec |
| 4 | 72ᵒC | 90sec |
| 5 | 72ᵒC | 7min | 1 |

PCR amplification of samples was confirmed by DNA electrophoresis using 1% w/v agarose (Sigma) gels. DNA was then precipitated and digested with the MspI restriction enzyme in NEBuffer 4 (1333U/ml, New England Biolabs) at 37ᵒC for 4 hours. Reactions were terminated at 65ᵒC (10mins) and dialysed on VSWP filters (Millipore) before mixture with GeneScan-500 Size Standard (Applied Biosystems) and subsequent sequencing on a 96-capillary 3730 DNA analyzer (Applied Biosystems/Hitachi, University of Chicago comprehensive cancer center).

Individual fragment lengths were then determined relative to the GeneScan-500 Size Standard and calculated using Gene Mapper® v4.1 software (Applied Biosystems). The additive main effects and multiplicative interaction model (AMMI, doubly-centered principal component analysis (PCA)) was then applied to the dataset using T-REX freeware (trex.biohpc.org) to generate PCA plots.

**Lymphocyte extraction from whole blood**

Adult murine lymphocytes were isolated from whole blood collected from cardiac laceration based on methods previously described with slight modification[^2^](#_ENREF_2). Briefly, blood was pooled from three mice and diluted 1:1 v/v in PBS-EDTA (2mM). 6ml of diluted blood was then layered on 4.5ml room temperature Ficoll-Paque Plus™ (GE healthcare) and the interphase between the plasma and Ficoll-Paque Plus™ solution enriched in lymphocytes was removed post-centrifugation (400xg, 35min, 18ᵒC, no braking). Lymphocytes were then washed twice in HBSS and used in downstream applications.

**RNA isolation and Q-PCR**

RNA from tissues was isolated using Trizol® (Life Technologies) according to manufacturer’s guidelines. For spleens only, Trizol-extracted RNA samples were then further purified using subsequent acid:phenol:choloroform:IAA (Ambion) isolation. Residual genomic DNA was then removed from the samples by DNase treatment using the TURBO DNA-free™ kit (Ambion) according to manufacturer’s guidelines. Total RNA yield and purity was then quantified by Nanodrop Lite (Thermo Fisher) and 2100 RNA Bioanalyzer (Agilent Technologies) assessment. A total of 1µg of sample RNA was then reverse transcribed to yield cDNA using a high capacity cDNA reverse transcription kit (Applied Biosystems) according to the manufacturer’s guidelines. Sample cDNA was then diluted 1:3 in DEPC-treated H_2_O prior to Q-PCR.

Q-PCR was conducted in 386-well plates on a Lightcycler® 480 thermal cycler (Roche) and all reactions were performed in triplicate. Primers used for SYBR green reactions were: For Taqman probe-based detection, genes were amplified using the SsoAdvanced™ universal probes supermix (BioRad). Taqman probes used in this study were: GAPDH (Mm99999915_g1), IFNβ (Mm00439552_s1), IFIT-1 (Mm00515153_m1), IRF-7 (Mm00516793_g1), CCL11 (Mm00441238_m1), IL-1β (Mm00434228_m1), TNFα (Mm00443258_m1). Gene expression was quantified using a modified ΔΔCt method[^3^](#_ENREF_3). Triplicate Ct values for genes of interest were normalised to Ct values of the GAPDH housekeeping gene (ΔCt). Data is then displayed as the inverse of this normalized value (1/ΔCt).

**Apolipoprotein E (ApoE) enzyme-linked immunosorbent assay (ELISA)**

For murine ApoE ELISA, plates were coated with monoclonal HJ6.2 antibodies (in-house generated) at a concentration of 10 μg/ml in ELISA coating buffer (0.05M Sodium carbonate-bicarbonate, pH9.6) at 4 °C overnight. After blocking with 2% w/v BSA in PBS at 37 °C for 1hr, the samples were loaded into 96-well plates in duplicate and incubated overnight at 4 °C. The plates were incubated in 300 ng/ml HJ6.8-biotin (generated in-house) at 37 °C for 1.5hr. Followed by an incubation in 1:10,000 Streptavidin Poly-HRP40 Conjugate (Fitzgerald) at room temperature for 1.5hr, the plates were developed using Super Slow ELISA TMB substrate (Sigma) and read on a Synergy™ 2 multi-mode plate reader at 650 nm (BioTek). Pooled plasma from C57BL/6J mice was used as a standard for murine ApoE quantification.

**SDS-PAGE, Western blotting and densitometry**

For Western blot analysis, brain tissue was ground in LN_2_ and homgenised in radioimmunoprecipitation assay (RIPA) buffer (50mM Tris, 150mM NaCl, 0.1% w/v SDS, 0.5% w/v sodium deoxycholate, 1% v/v Triton X-100) with protease and phosphatase inhibitors (Roche) via sonication. Samples were rotated at 4ᵒC for 2hr, centrifuged (13,000g, 5min, 4ᵒC), and protein concentration of the supernatant was determined by BCA assay (Thermo Fisher).

For SDS-PAGE, samples were denatured at 95ᵒC in reducing buffer (20mM Tris, 20%v/v glycerol, 4%w/v SDS, 10% β-mecaptoethanol, and bromophenol blue) prior to loading 25µg of protein onto 10% SDS-PAGE gels (60mM Tris, 0.1% w/v SDS, 0.1% w/v APS, 0.01% v/v TEMED, 10% Acrylamide/Bis). Gel electrophoresis was conducted in SDS-PAGE running buffer (Amresco) and then a wet transfer in Tris-glycine buffer (Amresco) onto a 0.2µm nitrocellulose membrane (BioRad) was performed. Membranes were then blocked in 5% w/v non-fat milk powder in PBS-Tween-20 (0.05% v/v, PBS-T) for 1hr at room temperature and then incubated with primary antibodies diluted in 2% w/v non-fat milk powder in PBS-T overnight at 4ᵒC (BioRad). Upon washing with PBS-T membranes were then incubated with HRP-conjugated secondary antibodies for 1hr at room temperature and chemiluminescent signals were produced by incubation with Western Lightning® Plus ECL (PerkinElmer). Chemiluminescent images were captured by exposure to x-ray film (MidSci) and subsequent photographic development (Kodak). Primary antibodies used were a mouse monoclonal anti-APP (26D6, 16pg/ml final concentration, in-house purified) and a mouse monoclonal anti-β-actin (1:40,000, A5441, Sigma). The secondary antibody used was an HRP-conjugated goat anti-mouse IgG (1:5000, 31430, Thermo Fisher).

For densitometry, X-ray film images were scanned and imported into ImageJ (NIH) as 8-Bit images. Background-normalised pixel intensities of bands of interest were calculated in arbitrary units and expressed relative to that of the β-actin loading control.

**Supplementary references**

1 Wang, Y. *et al.* 16S rRNA gene-based analysis of fecal microbiota from preterm infants with and without necrotizing enterocolitis. *The ISME journal* **3**, 944-954, doi:10.1038/ismej.2009.37 (2009).

2 Boyum, A. Isolation of mononuclear cells and granulocytes from human blood. Isolation of monuclear cells by one centrifugation, and of granulocytes by combining centrifugation and sedimentation at 1 g. *Scandinavian journal of clinical and laboratory investigation. Supplementum* **97**, 77-89 (1968).

3 Livak, K. J. & Schmittgen, T. D. Analysis of relative gene expression data using real-time quantitative PCR and the 2(-Delta Delta C(T)) Method. *Methods* **25**, 402-408, doi:10.1006/meth.2001.1262 (2001).


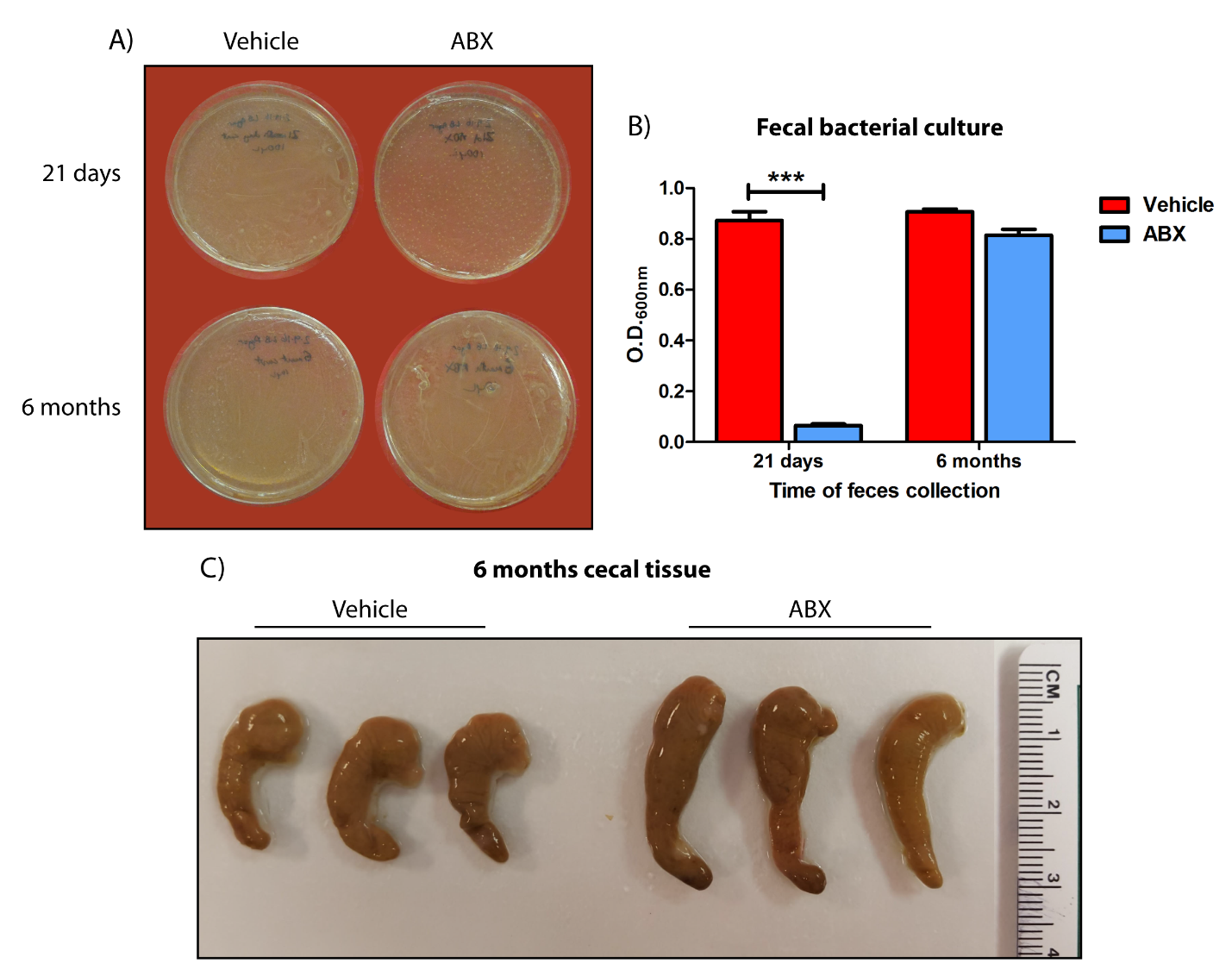


**Supplementary Figure 1. Bacterial growth monitoring from fecal homogenates of ABX-treated and vehicle control male APP_SWE_/PS1_ΔE9_ mice.**

**A)** Feces obtained from day 21 (post-gavage) and 6 month old (time of cull) male APP_SWE_/PS1_ΔE9_ mice treated with ABX or vehicle were homogenized in LB medium and spread on LB agar plates. Bacterial colony formation from fecal pellets is impeded in ABX-treated animals post-gavage but returns to vehicle-treated levels by the time of cull. **B)** Fecal homogenates from these animals were also cultured in LB media and optical density (O.D.) of cultures was measured at 600nm. Bacterial growth was significantly reduced in ABX-treated animals from fecal pellets taken at day 21 but returned to vehicle control levels at 6 months of age (*n*=6, ***p<0.0001, unpaired two-tailed Student’s *t*-test). **C)** Enlargement of the cecum, removed at 6 months of age, is evident in ABX-treated animals compared to vehicle controls. Data are displayed as mean ± SEM. See statistical table 1 for additional information.

**
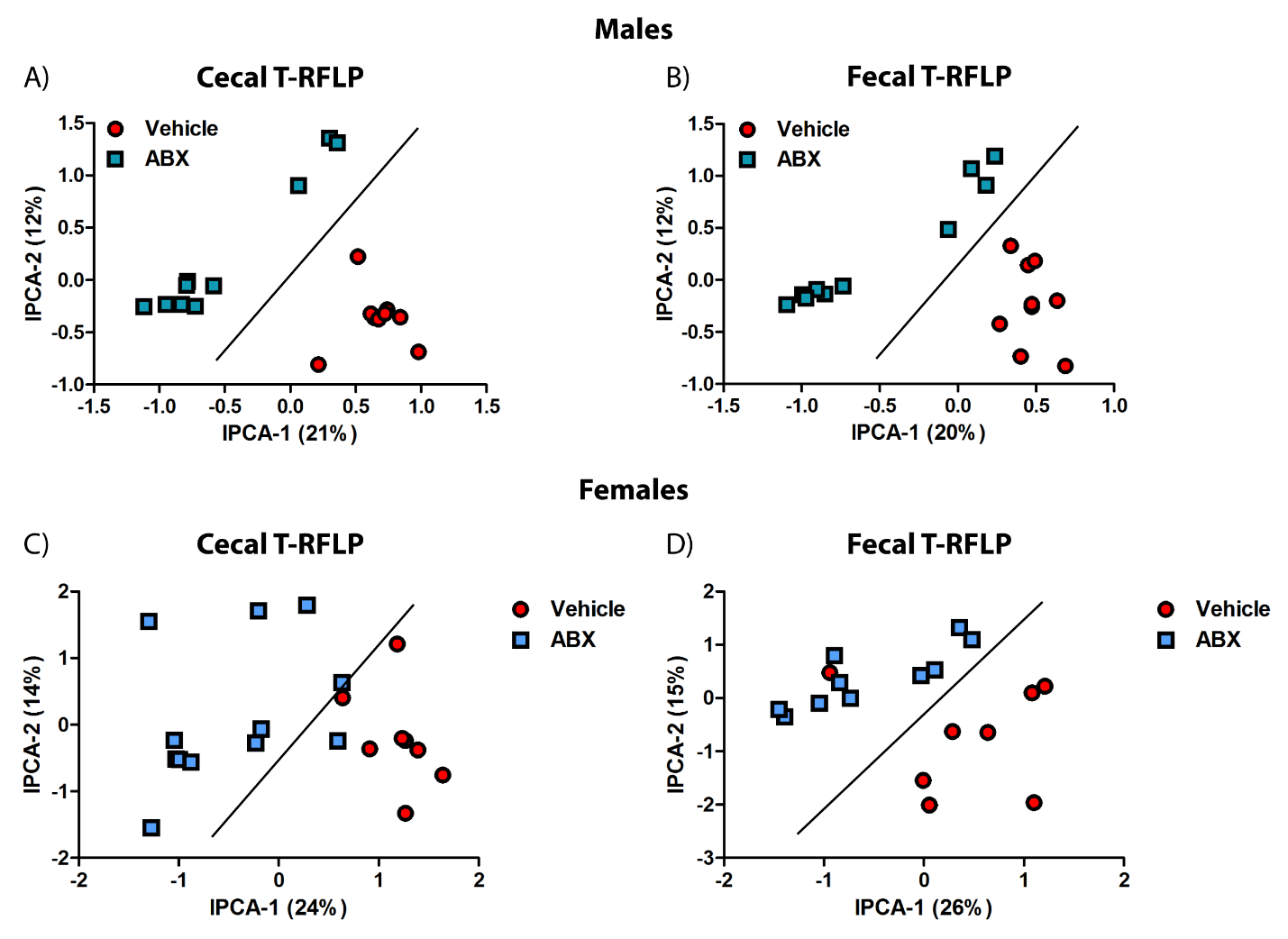
**

**Supplementary Figure 2. Principal co-ordinate analysis plots of 16s rRNA T-RFLP analysis in female APP_SWE_/PS1_ΔE9_ mice.**

**A)** T-RFLP analysis of the PCR-amplified 16s rRNA gene obtained from cecal contents of 6 month old male APP_SWE_/PS1_ΔE9_ mice (*n*=9-10). **B)** T-RFLP analysis of the PCR-amplified 16s rRNA gene obtained from fecal contents of 6 month old male APP_SWE_/PS1_ΔE9_ mice (*n*=9-10).**C)** T-RFLP analysis of the PCR-amplified 16s rRNA gene obtained from cecal contents of 5 month old female APP_SWE_/PS1_ΔE9_ mice. (*n*=8-10). **D)** T-RFLP analysis of the PCR-amplified 16s rRNA gene obtained from fecal contents of 5 month old female APP_SWE_/PS1_ΔE9_ mice (*n*=8-10). Principal co-ordinate analysis of DNA fragment lengths confirmed that ABX treatment induces alterations in microbial diversity. The percentage of data variance explained by each IPCA for all T-RFLP analysis is displayed. Data are displayed as X/Y scatter. See statistical table 1 for additional information.


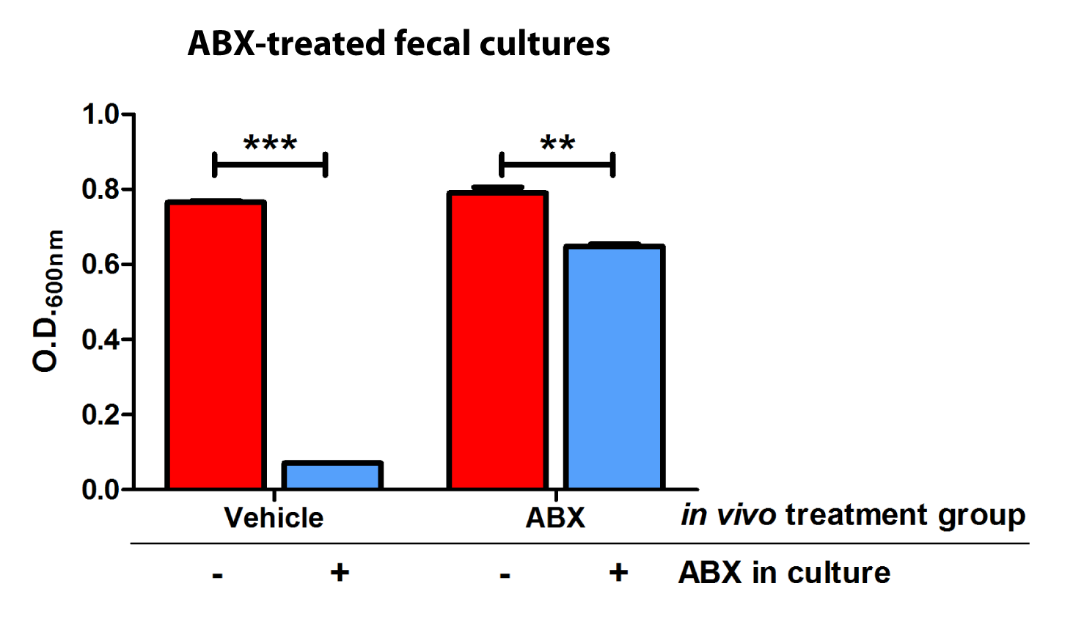


**Supplementary Figure 3. Effect of *ex-vivo* ABX-treatment on fecal homogenate bacterial cultures from ABX-treated and vehicle control male APP_SWE_/PS1_ΔE9_ mice.**

Fecal homogenates generated from 6 month old ABX-treated and vehicle control male APP_SWE_/PS1_ΔE9_ mice were cultured in LB media with and without ABX (supplied at *in-vivo* drinking water concentration). Analysis of the O.D._600nm_ of these cultures confirms that cultures derived from vehicle control animals are susceptible to ABX-induced toxicity however cultures derived from ABX-treated animals are largely resistant (*n*=6, **p=0.0047, ***p<0.0001, unpaired two-tailed Student’s *t*-test). Data are displayed as mean ± SEM. See statistical table 1 for additional information.


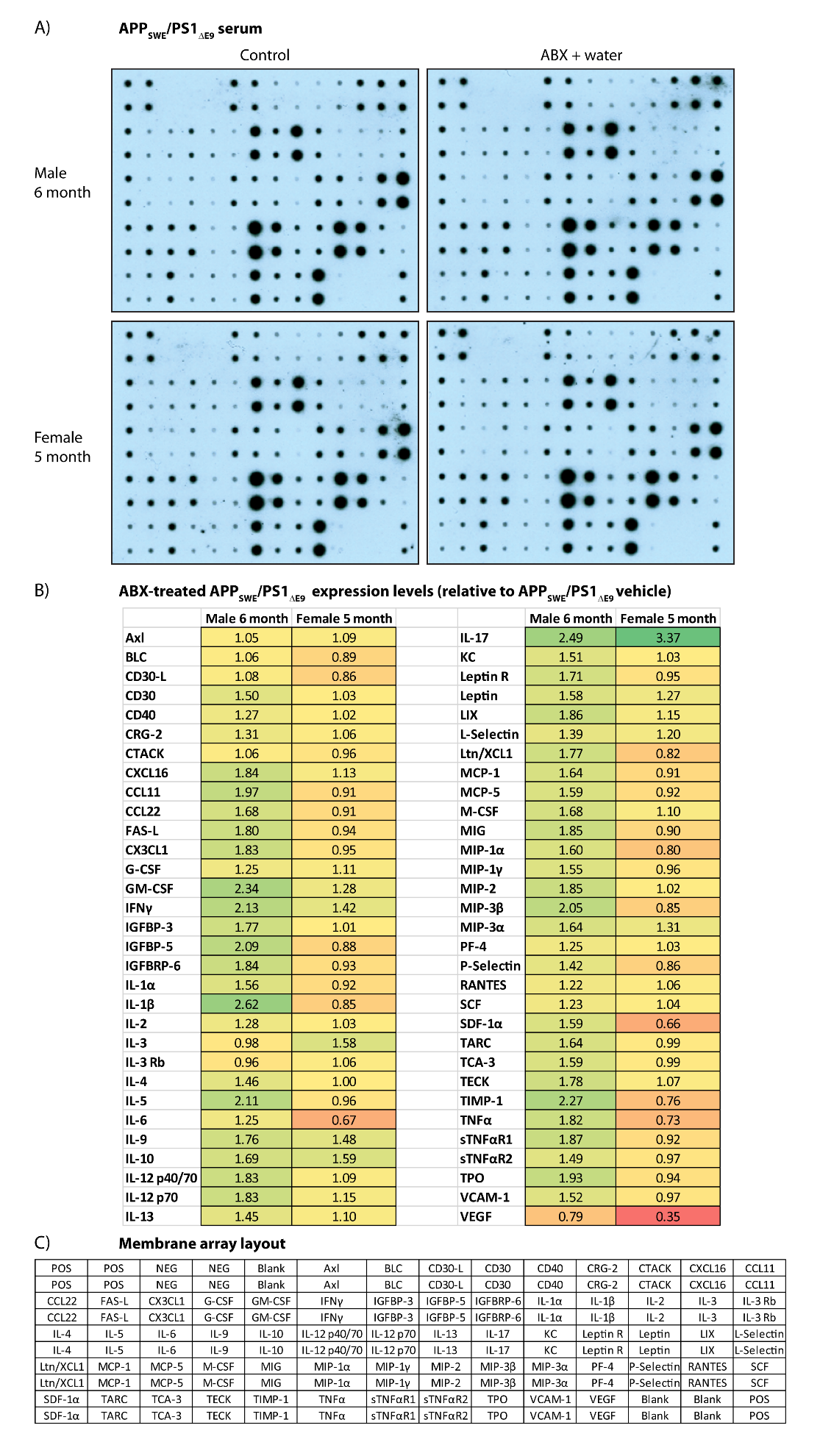


**Supplementary Figure 4. Immunoblot-based array of circulating inflammatory mediators in the sera of APP_SWE_/PS1_ΔE9_ mice.**

**A)** Immunoblot-based array of inflammatory mediators in isolated serum from vehicle control and ABX-treated male 6 month old (*n*=10, pooled sera) or female 5 month old (*n*=8-10, pooled sera) APP_SWE_/PS1_ΔE9_ mice. **B)** Densitometry of all 64 measured inflammatory mediator expression in ABX-treated APP_SWE_/PS1_ΔE9_ mice relative to vehicle control. Data was then stratified on a heat map whereby green represents up-regulation and red represented down-regulated expression relative to vehicle control. **C)** The dot-printed membrane array layout is shown.


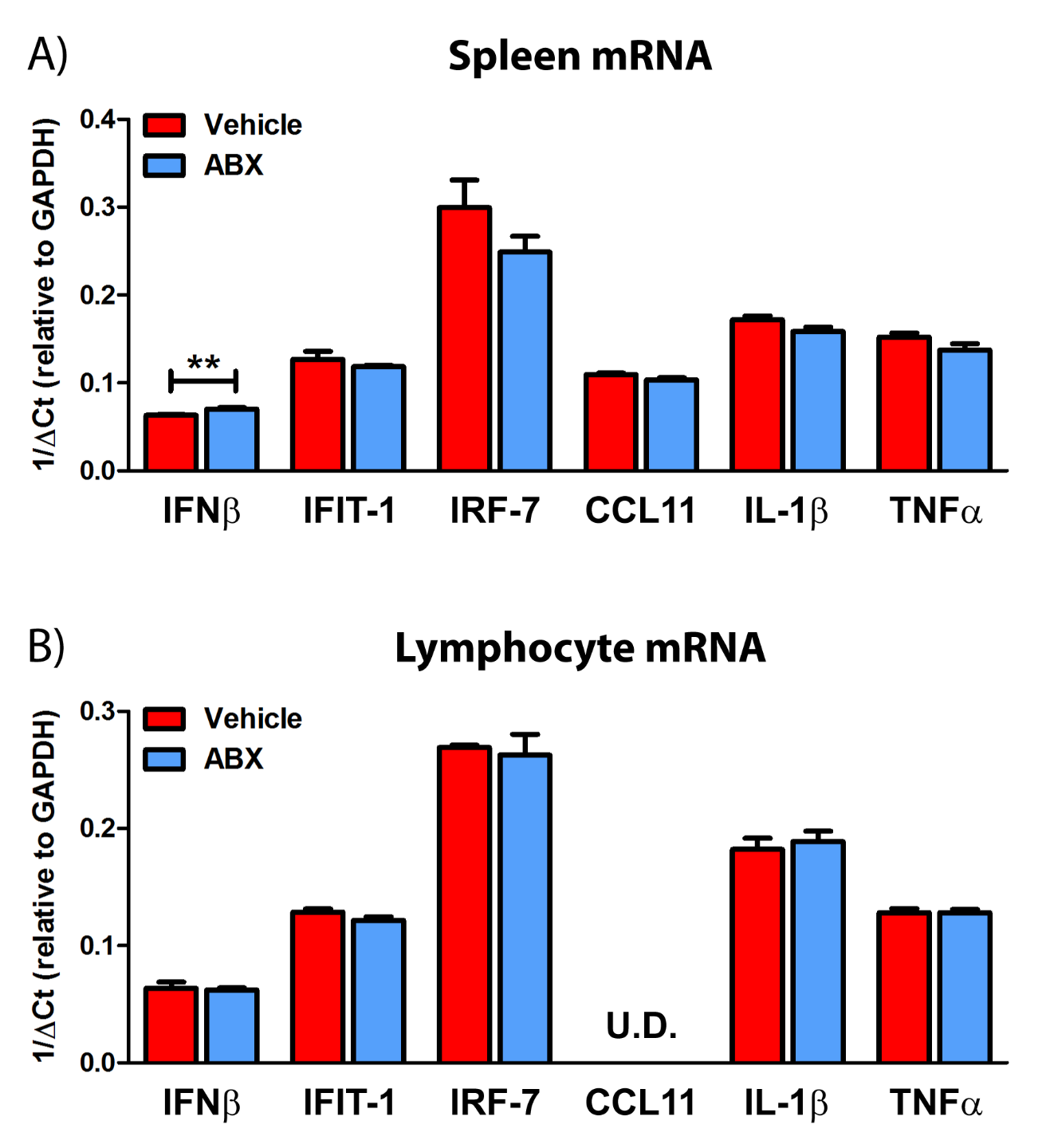


**Supplementary Figure 5. Q-PCR of inflammatory mediators in splenic tissue and isolated blood lymphocytes from male 6 month old vehicle and ABX-treated APP_SWE_/PS1_ΔE9_ mice.**

**A)** Q-PCR of splenic tissue isolated from vehicle and ABX-treated male 6 month old APP_SWE_/PS1_ΔE9_ mice analysing IFNβ, IFIT-1, IRF-7, CCL11, IL-1β and TNFα mRNA transcript levels (*n*=5, **p=0.0091, unpaired two-tailed Student’s *t*-test). **B)** Q-PCR of isolated circulating blood lymphocyte populations from the same treatment groups analysing expression levels of aforementioned genes (*n*=3, each sample contains pooled lymphocytes from 3 individual mice). CCL11 was undetectable (U.D.) in these samples. Data are displayed as mean ± SEM. See statistical table 1 for additional information.


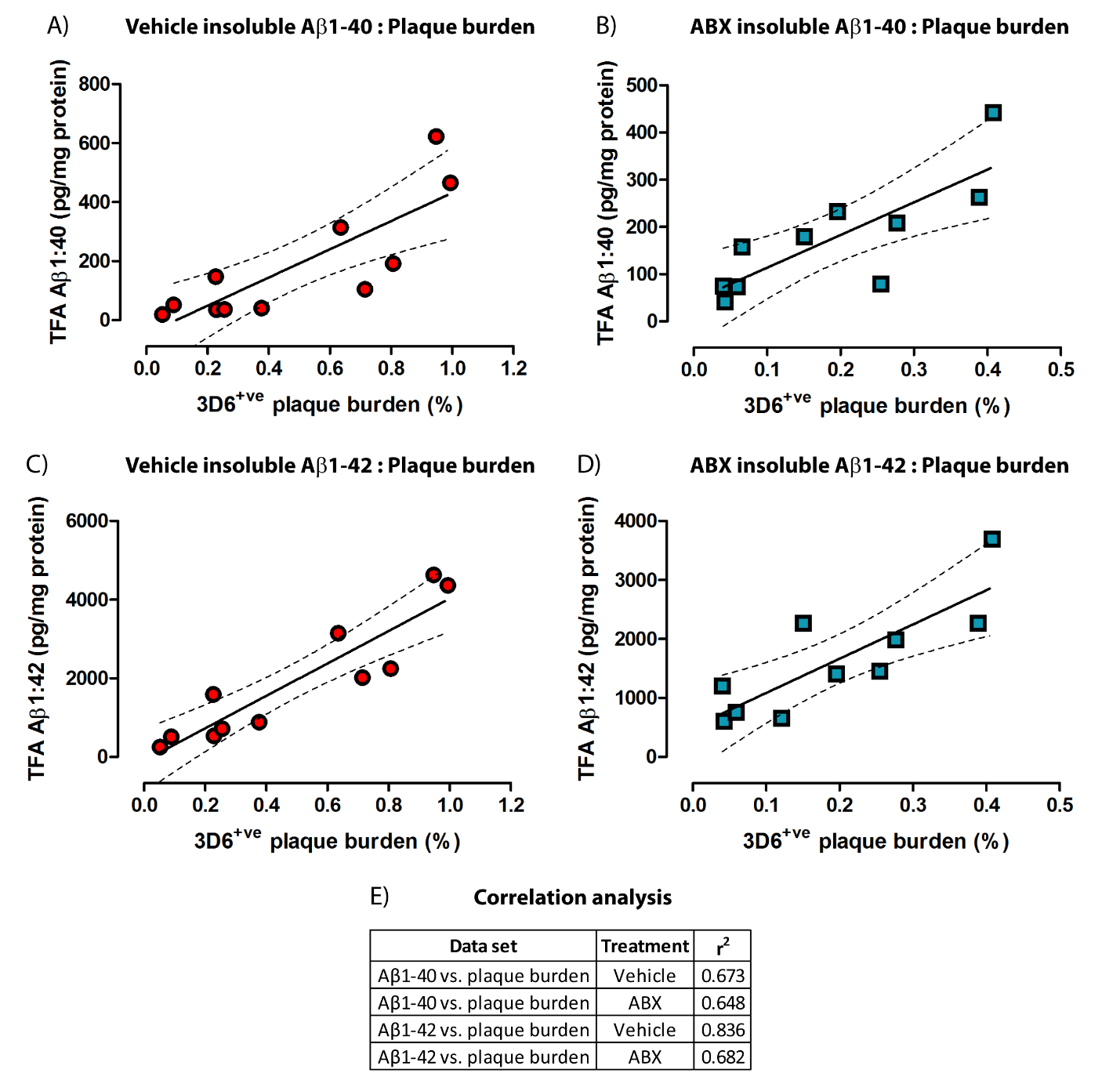


**Supplementary Figure 6. Analysis of correlation between TFA-soluble Aβ levels and Aβ plaques in male 6 month old APP_SWE_/PS1_ΔE9_ mice.**

TFA-soluble (Tris-insoluble) levels of Aβ1:40 and Aβ1:42 were determined by MSD Mesoscale® analysis using anti-Aβ mAb 4G8 capture antibody in vehicle control and ABX-treated 6 month old male APP_SWE_/PS1_ΔE9_ mice. The opposing hemisphere of these same mice were subjected to serial sectioning and immunohistochemistry using anti-Aβ mAb 3D6 to determine plaque burden. **A)** Pearson’s correlation plot of Aβ1:40 vs. plaque burden in vehicle control APP_SWE_/PS1_ΔE9_ mice. **B)** Pearson’s correlation plot of Aβ1:40 vs. plaque burden in ABX-treated APP_SWE_/PS1_ΔE9_ mice. **C)** Pearson’s correlation plot of Aβ1:42 vs. plaque burden in vehicle control APP_SWE_/PS1_ΔE9_ mice. **D)** Pearson’s correlation plot of Aβ1:42 vs. plaque burden in ABX-treated APP_SWE_/PS1_ΔE9_ mice. **E)** Calculated r^2^ correlation values from Pearson’s linear regression analysis for all datasets (*n*=10). Data are displayed as X/Y scatter with linear line of best fit and 95% confidence interval calculated by Pearson’s linear regression analysis. See statistical table 1 for additional information.


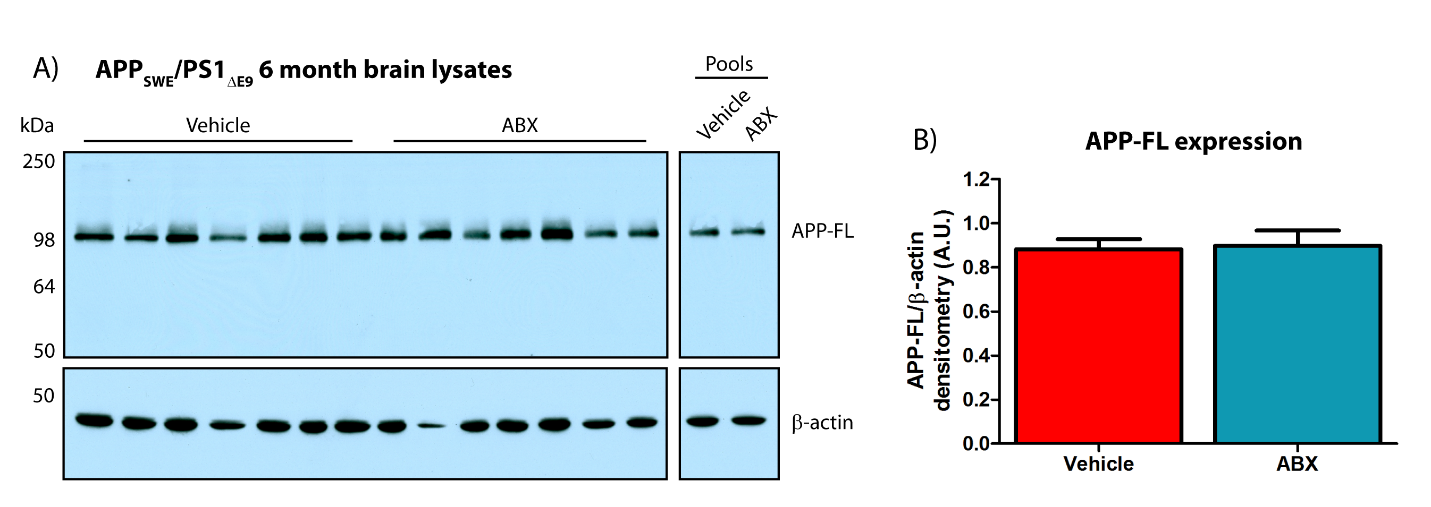


**Supplementary Figure 7. Expression of the APP transgene in ABX-treated and vehicle control in male 6 month old APP_SWE_/PS1_ΔE9_ mice**.

**A)** Immunoblot of full length APP (APP-FL) expression in RIPA-soluble brain lysates of vehicle control and ABX-treated male 6 month old APP_SWE_/PS1_ΔE9_ mice using anti-APP mAb 26D6. Samples were run both individually and as pools to confirm expression. **B)** Densitometry of APP-FL expression as detected by immunoblotting (*n*=7). All densitometry is expressed as a ratio of APP-FL:β-actin raw pixel intensities. Immuno-detection of β-actin was used to ascertain loading quantities. Data are displayed as mean ± SEM. See statistical table 1 for additional information.


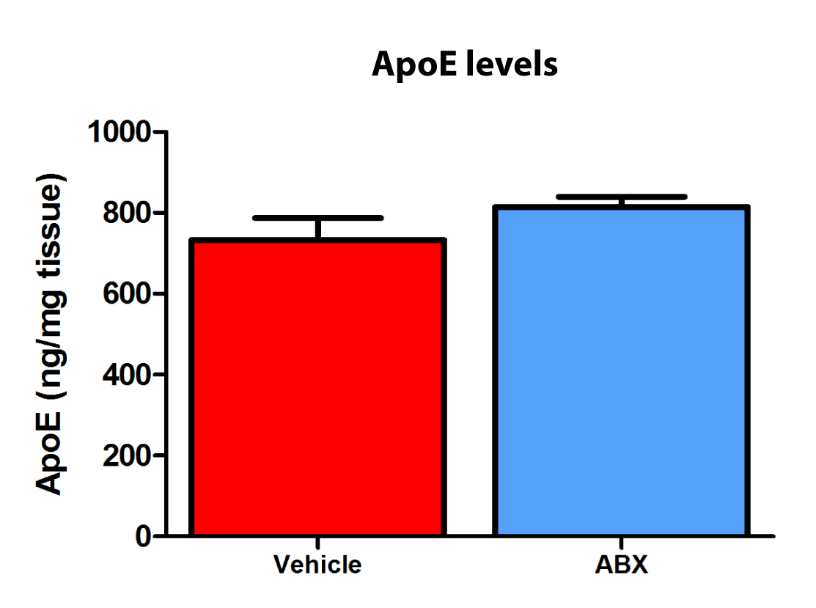


**Supplementary Figure 8. Brain ApoE levels in ABX-treated and vehicle control in male 6 month old APP_SWE_/PS1_ΔE9_ mice**.

Total ApoE levels within cortical and hippocampal tissue of vehicle control and ABX-treated male 6 month old APP_SWE_/PS1_ΔE9_ mice detected by ELISA (*n*=10). For all MSD Mesoscale® and ApoE ELISAs, concentrations were normalized to total protein concentration of combined cortical and hippocampal lysates as determined by BCA assay. Data are displayed as mean ± SEM. See statistical table 1 for additional information.


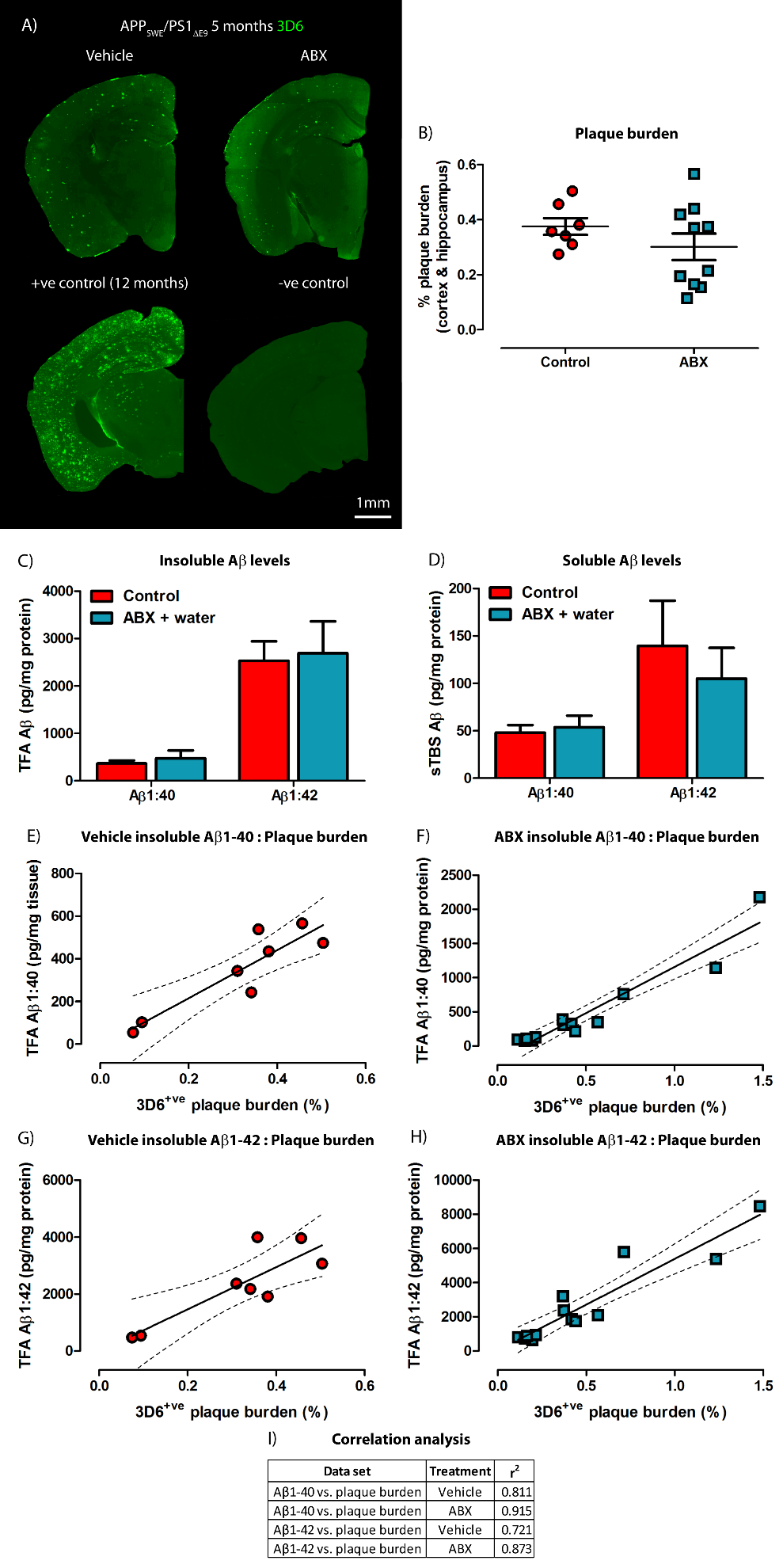


**Supplementary Figure 9. Effect of ABX treatment on amyloidosis in 5 month old female APP_SWE_/PS1_ΔE9_ mice.**

**A)** Representative immunohistochemical images of Aβ plaque deposition in vehicle control and ABX-treated female 5 month old APP_SWE_/PS1_ΔE9_ mice using anti-Aβ mAb 3D6. Each staining run was performed using sections from 12 month old APP_SWE_/PS1_ΔE9_ mice as a positive control and no primary antibody negative controls. **B)** Plaque burden quantification in vehicle control and ABX-treated female 5 month old APP_SWE_/PS1_ΔE9_ mice using particle analysis of 3D6^+ve^ immunofluorescence (*n*=8-10). 3D6^+ve^ area was averaged from 4 sections/mouse (240µm apart) and expressed relative to total cortical and hippocampal area. **C)** MSD Mesoscale® analysis of TFA-soluble (TBS-insoluble) Aβ1-40 and Aβ1-42 levels in combined cortical and hippocampal tissue of vehicle control and ABX-treated female 5 month old APP_SWE_/PS1_ΔE9_ mice using anti-Aβ mAb 4G8 (*n*=8-10). **D)** MSD Mesoscale® analysis of TBS-soluble Aβ1-40 and Aβ1-42 levels in combined cortical and hippocampal tissue of vehicle control and ABX-treated female 5 month old APP_SWE_/PS1_ΔE9_ mice using anti-Aβ mAb 4G8 (*n*=8-10). **E)** Pearson’s correlation plot of Aβ1:40 vs. plaque burden in vehicle control APP_SWE_/PS1_ΔE9_ mice. **F)** Pearson’s correlation plot of Aβ1:40 vs. plaque burden in ABX-treated APP_SWE_/PS1_ΔE9_ mice. **G)** Pearson’s correlation plot of Aβ1:42 vs. plaque burden in vehicle control APP_SWE_/PS1_ΔE9_ mice. **H)** Pearson’s correlation plot of Aβ1:42 vs. plaque burden in ABX-treated APP_SWE_/PS1_ΔE9_ mice. **I)** Calculated r^2^ correlation values from Pearson’s linear regression analysis for all datasets (*n*=8-10). Data are displayed as X/Y scatter with linear line of best fit and 95% confidence interval calculated by Pearson’s linear regression analysis. See statistical table 1 for additional information.

Supplementary table 1. Statistical analysis performed on all data sets presented in the current study.

| **Figure** | **Panel** | **Data structure** | **Statistical test** | **Power** | **Comparison** | ***p* value/r^2^ value** | ***N*** |
| --- | --- | --- | --- | --- | --- | --- | --- |
| 1 | A | Simple 2 group | Unpaired two-tailed Student's *t*-test | 0.0500 | Vehicle vs. ABX | 0.9921 | Vehicle: 9 ABX: 10 |
|  | B | Simple 2 group | Unpaired two-tailed Student's *t*-test | 0.1674 | Vehicle vs. ABX | 0.3221 | Vehicle: 9 ABX: 10 |
|  | E | Simple 2 group | Unpaired two-tailed Student's *t*-test | 0.8184 | Vehicle vs. ABX | 0.0429 | Vehicle: 9 ABX: 10 |
| 3 | B | Simple 2 group | Unpaired two-tailed Student's *t*-test | 0.7334 | Vehicle vs. ABX | 0.0137 | 10 each treatment group |
|  | D | Simple 2 group | Unpaired two-tailed Student's *t*-test | 0.9152 | Vehicle vs. ABX | 0.0109 | 10 each treatment group |
|  | E | Simple 2 group (2 data sets on same axis) | Unpaired two-tailed Student's *t*-test | 0.1046 | Aβ1-40: Vehicle vs. ABX | 0.4418 | 10 each treatment group |
|  |  |  |  | 0.4017 | Aβ1-42: Vehicle vs. ABX | 0.0491 |  |
|  | F | Simple 2 group (2 data sets on same axis) | Unpaired two-tailed Student's *t*-test | 0.6098 | Aβ1-40: Vehicle vs. ABX | 0.0176 | 10 each treatment group |
|  |  |  |  | 0.5094 | Aβ1-42: Vehicle vs. ABX | 0.0326 |  |
|  | G | Simple 2 group | Unpaired two-tailed Student's *t*-test | 0.6800 | Vehicle vs. ABX | 0.014 | 10 each treatment group |
| 4 | B | Simple 2 group | Unpaired two-tailed Student's t-test | 0.9999 | Vehicle vs. ABX | <0.0001 | 8 each treatment group |
|  | C | Simple 2 group | Unpaired two-tailed Student's t-test | 0.0616 | Vehicle vs. ABX | 0.7478 | 8 each treatment group |
|  | E | Simple 2 group | Unpaired two-tailed Student's t-test | 0.8991 | Vehicle vs. ABX | 0.008 | 4 each treatment group |
|  | F | Simple 2 group | Unpaired two-tailed Student's t-test | 0.7099 | Vehicle vs. ABX | 0.0237 | 4 each treatment group |
|  | G | Simple 2 group | Unpaired two-tailed Student's t-test | 0.9159 | Vehicle vs. ABX | 0.0069 | 4 each treatment group |
|  | I | Simple 2 group | Unpaired two-tailed Student's t-test | 0.9574 | Vehicle vs. ABX | 0.002 | 8 each treatment group |
|  | J | Simple 2 group | Unpaired two-tailed Student's t-test | 0.0799 | Vehicle vs. ABX | 0.6157 | 8 each treatment group |
| S1 | B | Simple 2 group (2 data sets on same axis) | Unpaired two-tailed Student's t-test | 1.0000 | 21 days: Vehicle vs. ABX | <0.0001 | 6 each treatment group |
|  |  |  |  | 0.9022 | 6 months: Vehicle vs. ABX | 0.0515 | 6 each treatment group |
| S3 | N/A | Simple 2 group (2 data sets on same axis) | Unpaired two-tailed Student's t-test | 1.0000 | Vehicle culture: Vehicle vs. ABX | <0.0001 | 6 each treatment group |
|  |  |  |  | 0.9859 | ABX culture: Vehicle vs. ABX | 0.0012 | 6 each treatment group |
| S5 | A | Simple 2 group (6 data sets on same axis) | Unpaired two-tailed Student's t-test | IFNβ: 0.9266 IFIT-1: 0.1541 IRF-7: 0.2655 CCL11: 0.5098 IL-1β: 0.4733 TNFα: 0.3632 | Vehicle vs. ABX | IFNβ: 0.0091 IFIT-1: 0.3213 IRF-7: 0.1858 CCL11: 0.0821 IL-1β: 0.0870 TNFα: 0.1409 | 5 each treatment group |
|  | B | Simple 2 group (6 data sets on same axis) | Unpaired two-tailed Student's t-test | IFNβ: 0.0557 IFIT-1: 0.2604 IRF-7: 0.0597 CCL11: N/A IL-1β: 0.0691 TNFα: 0.0500 | Vehicle vs. ABX | IFNβ: 0.7899 IFIT-1: 0.1612 IRF-7: 0.7372 CCL11: N/A IL-1β: 0.6332 TNFα: 0.9934 | 3 each treatment group (each *n* represents pools from 3 mice) |
| S6 | A | X/Y scatter correlation | Pearson's linear regression | 0.6777 | Aβ1-40 vs. 3D6^+ve^ plaques | 0.6731 | 10 |
|  | B | X/Y scatter correlation | Pearson's linear regression | 0.6224 | Aβ1-42 vs. 3D6^+ve^ plaques | 0.6476 | 10 |
|  | C | X/Y scatter correlation | Pearson's linear regression | 0.9449 | Aβ1-40 vs. 3D6^+ve^ plaques | 0.8359 | 10 |
|  | D | X/Y scatter correlation | Pearson's linear regression | 0.6986 | Aβ1-42 vs. 3D6^+ve^ plaques | 0.6823 | 10 |
| S7 | B | Simple 2 group | Unpaired two-tailed Student's t-test | 0.0555 | Vehicle vs. ABX | 0.1631 | 7 each treatment group |
| S8 | N/A | Simple 2 group | Unpaired two-tailed Student's t-test | 0.2014 | Vehicle vs. ABX | 0.1768 | 10 each genotype |
| S9 | B | Simple 2 group | Unpaired two-tailed Student's t-test | 0.2523 | Vehicle vs. ABX | 0.2592 | Vehicle: 8 ABX: 10 |
|  | C | Simple 2 group (2 data sets on same axis) | Unpaired two-tailed Student's t-test | 0.0780 | Aβ1-40: Vehicle vs. ABX | 0.6238 | Vehicle: 8 ABX: 10 |
|  |  |  |  | 0.0531 | Aβ1-42: Vehicle vs. ABX | 0.8666 | Vehicle: 8 ABX: 10 |
|  | D | Simple 2 group (2 data sets on same axis) | Unpaired two-tailed Student's t-test | 0.0617 | Aβ1-40: Vehicle vs. ABX | 0.7429 | Vehicle: 8 ABX: 10 |
|  |  |  |  | 0.0844 | Aβ1-42: Vehicle vs. ABX | 0.542 | Vehicle: 8 ABX: 10 |
|  | E | X/Y scatter correlation | Pearson's linear regression | 0.9002 | Aβ1-40 vs. 3D6^+ve^ plaques | 0.8105 | 8 |
|  | F | X/Y scatter correlation | Pearson's linear regression | 0.9994 | Aβ1-42 vs. 3D6^+ve^ plaques | 0.9145 | 10 |
|  | G | X/Y scatter correlation | Pearson's linear regression | 0.6908 | Aβ1-40 vs. 3D6^+ve^ plaques | 0.7211 | 8 |
|  | H | X/Y scatter correlation | Pearson's linear regression | 0.9859 | Aβ1-42 vs. 3D6^+ve^ plaques | 0.8729 | 10 |

**Supplementary table 2. Complete OTU read data from 16s rRNA gene sequencing of cecal and fecal contents from 6 month old male APP_SWE_/PS1_ΔE9_ mice (related to figure 1).**

| **Taxonomy** | **Proportion of total OTU reads (%)** | |
| --- | --- | --- |
|  | **Treatment** | |
|  | **Vehicle** | **ABX** |
| k__Archaea;p__Euryarchaeota;c__Methanomicrobia;o__Methanosarcinales;f__Methanosarcinaceae;g__Methanosarcina | 0.0000000 | 0.0000611 |
| k__Bacteria;p__Acidobacteria;c__Solibacteres;o__Solibacterales;f__Solibacteraceae;g__Candidatus Solibacter | 0.0000000 | 0.0000611 |
| k__Bacteria;p__Actinobacteria;c__Actinobacteria;o__Actinomycetales;Other;Other | 0.0000190 | 0.0000306 |
| k__Bacteria;p__Actinobacteria;c__Actinobacteria;o__Actinomycetales;f__Brevibacteriaceae;g__Brevibacterium | 0.0000379 | 0.0001222 |
| k__Bacteria;p__Actinobacteria;c__Actinobacteria;o__Actinomycetales;f__Corynebacteriaceae;g__Corynebacterium | 0.0007777 | 0.0010391 |
| k__Bacteria;p__Actinobacteria;c__Actinobacteria;o__Actinomycetales;f__Dermabacteraceae;g__Brachybacterium | 0.0000000 | 0.0000917 |
| k__Bacteria;p__Actinobacteria;c__Actinobacteria;o__Actinomycetales;f__Gordoniaceae;g__ | 0.0000000 | 0.0001222 |
| k__Bacteria;p__Actinobacteria;c__Actinobacteria;o__Actinomycetales;f__Microbacteriaceae;Other | 0.0002276 | 0.0007946 |
| k__Bacteria;p__Actinobacteria;c__Actinobacteria;o__Actinomycetales;f__Microbacteriaceae;g__Microbacterium | 0.0000379 | 0.0002139 |
| k__Bacteria;p__Actinobacteria;c__Actinobacteria;o__Actinomycetales;f__Microbacteriaceae;g__Salinibacterium | 0.0000000 | 0.0000611 |
| k__Bacteria;p__Actinobacteria;c__Actinobacteria;o__Actinomycetales;f__Micrococcaceae;g__Arthrobacter | 0.0000190 | 0.0000306 |
| k__Bacteria;p__Actinobacteria;c__Actinobacteria;o__Actinomycetales;f__Micrococcaceae;g__Kocuria | 0.0000379 | 0.0000306 |
| k__Bacteria;p__Actinobacteria;c__Actinobacteria;o__Actinomycetales;f__Nocardiaceae;g__Rhodococcus | 0.0000569 | 0.0000611 |
| k__Bacteria;p__Actinobacteria;c__Actinobacteria;o__Actinomycetales;f__Nocardioidaceae;g__ | 0.0000000 | 0.0000000 |
| k__Bacteria;p__Actinobacteria;c__Actinobacteria;o__Actinomycetales;f__Nocardiopsaceae;Other | 0.0000000 | 0.0000611 |
| k__Bacteria;p__Actinobacteria;c__Actinobacteria;o__Actinomycetales;f__Promicromonosporaceae;g__Cellulosimicrobium | 0.0003793 | 0.0000000 |
| k__Bacteria;p__Actinobacteria;c__Actinobacteria;o__Actinomycetales;f__Propionibacteriaceae;g__Propionibacterium | 0.0000000 | 0.0000611 |
| k__Bacteria;p__Actinobacteria;c__Actinobacteria;o__Actinomycetales;f__Streptomycetaceae;Other | 0.0000000 | 0.0001222 |
| k__Bacteria;p__Actinobacteria;c__Actinobacteria;o__Actinomycetales;f__Yaniellaceae;g__Yaniella | 0.0000000 | 0.0000611 |
| k__Bacteria;p__Actinobacteria;c__Actinobacteria;o__Bifidobacteriales;Other;Other | 0.0000190 | 0.0000000 |
| k__Bacteria;p__Actinobacteria;c__Actinobacteria;o__Bifidobacteriales;f__Bifidobacteriaceae;g__Bifidobacterium | 0.0823947 | 0.0009474 |
| k__Bacteria;p__Actinobacteria;c__Thermoleophilia;o__Solirubrobacterales;Other;Other | 0.0000190 | 0.0000306 |
| k__Bacteria;p__Bacteroidetes;Other;Other;Other;Other | 0.0990671 | 0.0318452 |
| k__Bacteria;p__Bacteroidetes;c__Bacteroidia;Other;Other;Other | 0.0005121 | 0.0000611 |
| k__Bacteria;p__Bacteroidetes;c__Bacteroidia;o__Bacteroidales;Other;Other | 7.4351540 | 0.1514941 |
| k__Bacteria;p__Bacteroidetes;c__Bacteroidia;o__Bacteroidales;f__;g__ | 4.7711157 | 1.8820168 |
| k__Bacteria;p__Bacteroidetes;c__Bacteroidia;o__Bacteroidales;f__Bacteroidaceae;Other | 0.0239749 | 0.0064179 |
| k__Bacteria;p__Bacteroidetes;c__Bacteroidia;o__Bacteroidales;f__Bacteroidaceae;g__Bacteroides | 4.7273388 | 3.6852456 |
| k__Bacteria;p__Bacteroidetes;c__Bacteroidia;o__Bacteroidales;f__Porphyromonadaceae;Other | 0.0073594 | 0.0009168 |
| k__Bacteria;p__Bacteroidetes;c__Bacteroidia;o__Bacteroidales;f__Porphyromonadaceae;g__Parabacteroides | 3.8229698 | 0.9864994 |
| k__Bacteria;p__Bacteroidetes;c__Bacteroidia;o__Bacteroidales;f__Prevotellaceae;Other | 0.0382764 | 0.0177258 |
| k__Bacteria;p__Bacteroidetes;c__Bacteroidia;o__Bacteroidales;f__Prevotellaceae;g__Prevotella | 2.8286190 | 2.2337813 |
| k__Bacteria;p__Bacteroidetes;c__Bacteroidia;o__Bacteroidales;f__Rikenellaceae;Other | 0.1416301 | 0.0040341 |
| k__Bacteria;p__Bacteroidetes;c__Bacteroidia;o__Bacteroidales;f__Rikenellaceae;g__ | 2.7989918 | 0.5555191 |
| k__Bacteria;p__Bacteroidetes;c__Bacteroidia;o__Bacteroidales;f__Rikenellaceae;g__AF12 | 0.2013776 | 0.0008863 |
| k__Bacteria;p__Bacteroidetes;c__Bacteroidia;o__Bacteroidales;f__Rikenellaceae;g__Rikenella | 0.0000948 | 0.0000000 |
| k__Bacteria;p__Bacteroidetes;c__Bacteroidia;o__Bacteroidales;f__S24-7;g__ | 27.8169730 | 11.0720873 |
| k__Bacteria;p__Bacteroidetes;c__Bacteroidia;o__Bacteroidales;f__[Odoribacteraceae];Other | 0.0001707 | 0.0000000 |
| k__Bacteria;p__Bacteroidetes;c__Bacteroidia;o__Bacteroidales;f__[Odoribacteraceae];g__Odoribacter | 1.5111195 | 0.0067847 |
| k__Bacteria;p__Bacteroidetes;c__Bacteroidia;o__Bacteroidales;f__[Paraprevotellaceae];Other | 0.0003224 | 0.0000000 |
| k__Bacteria;p__Bacteroidetes;c__Bacteroidia;o__Bacteroidales;f__[Paraprevotellaceae];g__Paraprevotella | 0.4470443 | 0.0014975 |
| k__Bacteria;p__Bacteroidetes;c__Bacteroidia;o__Bacteroidales;f__[Paraprevotellaceae];g__[Prevotella] | 0.0002466 | 0.0000306 |
| k__Bacteria;p__Chloroflexi;c__Chloroflexi;o__;f__;g__ | 0.0000190 | 0.0003362 |
| k__Bacteria;p__Cyanobacteria;Other;Other;Other;Other | 0.0007208 | 0.0000306 |
| k__Bacteria;p__Cyanobacteria;c__4C0d-2;Other;Other;Other | 0.0002086 | 0.0000000 |
| k__Bacteria;p__Cyanobacteria;c__4C0d-2;o__YS2;f__;g__ | 0.4109682 | 0.0690693 |
| k__Bacteria;p__Cyanobacteria;c__Chloroplast;o__Streptophyta;f__;g__ | 0.0022002 | 0.0090768 |
| k__Bacteria;p__Cyanobacteria;c__Synechococcophycideae;o__Pseudanabaenales;f__Pseudanabaenaceae;Other | 0.0001517 | 0.0003056 |
| k__Bacteria;p__Cyanobacteria;c__Synechococcophycideae;o__Pseudanabaenales;f__Pseudanabaenaceae;g__ | 0.0000000 | 0.0000917 |
| k__Bacteria;p__Cyanobacteria;c__Synechococcophycideae;o__Pseudanabaenales;f__Pseudanabaenaceae;g__Halomicronema | 0.0000190 | 0.0005501 |
| k__Bacteria;p__Deferribacteres;c__Deferribacteres;o__Deferribacterales;f__Deferribacteraceae;g__Mucispirillum | 0.8609525 | 0.0041564 |
| k__Bacteria;p__Firmicutes;Other;Other;Other;Other | 0.0996741 | 0.6469901 |
| k__Bacteria;p__Firmicutes;c__Bacilli;Other;Other;Other | 0.0086681 | 0.0039425 |
| k__Bacteria;p__Firmicutes;c__Bacilli;o__Bacillales;Other;Other | 0.0000569 | 0.0016809 |
| k__Bacteria;p__Firmicutes;c__Bacilli;o__Bacillales;f__Bacillaceae;Other | 0.0000379 | 0.0001222 |
| k__Bacteria;p__Firmicutes;c__Bacilli;o__Bacillales;f__Bacillaceae;g__Geobacillus | 0.0001138 | 0.0083739 |
| k__Bacteria;p__Firmicutes;c__Bacilli;o__Bacillales;f__Paenibacillaceae;g__Paenibacillus | 0.0000000 | 0.0000611 |
| k__Bacteria;p__Firmicutes;c__Bacilli;o__Bacillales;f__Planococcaceae;Other | 0.0000948 | 0.0000611 |
| k__Bacteria;p__Firmicutes;c__Bacilli;o__Bacillales;f__Planococcaceae;g__Sporosarcina | 0.0002086 | 0.0000000 |
| k__Bacteria;p__Firmicutes;c__Bacilli;o__Bacillales;f__Staphylococcaceae;Other | 0.0000948 | 0.0023532 |
| k__Bacteria;p__Firmicutes;c__Bacilli;o__Bacillales;f__Staphylococcaceae;g__Jeotgalicoccus | 0.0002655 | 0.0000000 |
| k__Bacteria;p__Firmicutes;c__Bacilli;o__Bacillales;f__Staphylococcaceae;g__Staphylococcus | 0.0027123 | 0.0796131 |
| k__Bacteria;p__Firmicutes;c__Bacilli;o__Lactobacillales;Other;Other | 0.0149464 | 0.0061123 |
| k__Bacteria;p__Firmicutes;c__Bacilli;o__Lactobacillales;f__Aerococcaceae;g__ | 0.0000379 | 0.0000000 |
| k__Bacteria;p__Firmicutes;c__Bacilli;o__Lactobacillales;f__Aerococcaceae;g__Aerococcus | 0.0004742 | 0.0000000 |
| k__Bacteria;p__Firmicutes;c__Bacilli;o__Lactobacillales;f__Enterococcaceae;Other | 0.0011191 | 0.0000000 |
| k__Bacteria;p__Firmicutes;c__Bacilli;o__Lactobacillales;f__Enterococcaceae;g__Enterococcus | 0.3312100 | 0.0018031 |
| k__Bacteria;p__Firmicutes;c__Bacilli;o__Lactobacillales;f__Lactobacillaceae;Other | 0.0970945 | 0.0712698 |
| k__Bacteria;p__Firmicutes;c__Bacilli;o__Lactobacillales;f__Lactobacillaceae;g__ | 2.3423500 | 2.2979302 |
| k__Bacteria;p__Firmicutes;c__Bacilli;o__Lactobacillales;f__Lactobacillaceae;g__Lactobacillus | 0.3985066 | 0.1322708 |
| k__Bacteria;p__Firmicutes;c__Bacilli;o__Lactobacillales;f__Streptococcaceae;Other | 0.0000000 | 0.0000000 |
| k__Bacteria;p__Firmicutes;c__Bacilli;o__Lactobacillales;f__Streptococcaceae;g__Streptococcus | 0.0409887 | 0.0012530 |
| k__Bacteria;p__Firmicutes;c__Bacilli;o__Turicibacterales;f__Turicibacteraceae;g__Turicibacter | 0.1156826 | 0.0007335 |
| k__Bacteria;p__Firmicutes;c__Clostridia;Other;Other;Other | 1.1150218 | 2.2488176 |
| k__Bacteria;p__Firmicutes;c__Clostridia;o__;f__;g__ | 0.1047194 | 0.0019559 |
| k__Bacteria;p__Firmicutes;c__Clostridia;o__Clostridiales;Other;Other | 0.1819739 | 0.0577310 |
| k__Bacteria;p__Firmicutes;c__Clostridia;o__Clostridiales;f__;g__ | 0.5265560 | 0.8185020 |
| k__Bacteria;p__Firmicutes;c__Clostridia;o__Clostridiales;f__Christensenellaceae;g__ | 0.0047039 | 0.0000306 |
| k__Bacteria;p__Firmicutes;c__Clostridia;o__Clostridiales;f__Clostridiaceae;Other | 0.0003793 | 0.0002445 |
| k__Bacteria;p__Firmicutes;c__Clostridia;o__Clostridiales;f__Clostridiaceae;g__ | 0.1415353 | 0.5388018 |
| k__Bacteria;p__Firmicutes;c__Clostridia;o__Clostridiales;f__Clostridiaceae;g__Clostridium | 0.0351278 | 0.0003056 |
| k__Bacteria;p__Firmicutes;c__Clostridia;o__Clostridiales;f__Dehalobacteriaceae;g__Dehalobacterium | 0.2422905 | 0.0017115 |
| k__Bacteria;p__Firmicutes;c__Clostridia;o__Clostridiales;f__Eubacteriaceae;g__Anaerofustis | 0.0076249 | 0.0025672 |
| k__Bacteria;p__Firmicutes;c__Clostridia;o__Clostridiales;f__Lachnospiraceae;Other | 5.1033120 | 12.5039310 |
| k__Bacteria;p__Firmicutes;c__Clostridia;o__Clostridiales;f__Lachnospiraceae;g__ | 11.3702974 | 16.3866965 |
| k__Bacteria;p__Firmicutes;c__Clostridia;o__Clostridiales;f__Lachnospiraceae;g__Anaerostipes | 0.0971893 | 0.0005501 |
| k__Bacteria;p__Firmicutes;c__Clostridia;o__Clostridiales;f__Lachnospiraceae;g__Blautia | 0.0000190 | 0.0000306 |
| k__Bacteria;p__Firmicutes;c__Clostridia;o__Clostridiales;f__Lachnospiraceae;g__Dorea | 0.2276666 | 0.0377436 |
| k__Bacteria;p__Firmicutes;c__Clostridia;o__Clostridiales;f__Lachnospiraceae;g__[Ruminococcus] | 0.5957114 | 1.9876378 |
| k__Bacteria;p__Firmicutes;c__Clostridia;o__Clostridiales;f__Peptostreptococcaceae;Other | 0.0007018 | 0.0000000 |
| k__Bacteria;p__Firmicutes;c__Clostridia;o__Clostridiales;f__Peptostreptococcaceae;g__ | 0.0498845 | 0.0012530 |
| k__Bacteria;p__Firmicutes;c__Clostridia;o__Clostridiales;f__Ruminococcaceae;Other | 1.0484649 | 1.1966107 |
| k__Bacteria;p__Firmicutes;c__Clostridia;o__Clostridiales;f__Ruminococcaceae;g__ | 1.5980474 | 0.0477679 |
| k__Bacteria;p__Firmicutes;c__Clostridia;o__Clostridiales;f__Ruminococcaceae;g__Faecalibacterium | 0.0000569 | 0.0000000 |
| k__Bacteria;p__Firmicutes;c__Clostridia;o__Clostridiales;f__Ruminococcaceae;g__Oscillospira | 4.8275250 | 3.3531016 |
| k__Bacteria;p__Firmicutes;c__Clostridia;o__Clostridiales;f__Ruminococcaceae;g__Ruminococcus | 1.3334322 | 0.2348663 |
| k__Bacteria;p__Firmicutes;c__Clostridia;o__Coriobacteriales;f__Coriobacteriaceae;Other | 0.0099390 | 0.0000306 |
| k__Bacteria;p__Firmicutes;c__Clostridia;o__Coriobacteriales;f__Coriobacteriaceae;g__ | 0.0046850 | 0.0000000 |
| k__Bacteria;p__Firmicutes;c__Clostridia;o__Coriobacteriales;f__Coriobacteriaceae;g__Adlercreutzia | 0.1044349 | 0.0007335 |
| k__Bacteria;p__Firmicutes;c__Erysipelotrichi;o__Erysipelotrichales;Other;Other | 0.0010432 | 0.0077015 |
| k__Bacteria;p__Firmicutes;c__Erysipelotrichi;o__Erysipelotrichales;f__Erysipelotrichaceae;Other | 0.0045332 | 0.0171451 |
| k__Bacteria;p__Firmicutes;c__Erysipelotrichi;o__Erysipelotrichales;f__Erysipelotrichaceae;g__ | 0.2426698 | 1.2835892 |
| k__Bacteria;p__Firmicutes;c__Erysipelotrichi;o__Erysipelotrichales;f__Erysipelotrichaceae;g__Allobaculum | 0.9776783 | 7.6035849 |
| k__Bacteria;p__Firmicutes;c__Erysipelotrichi;o__Erysipelotrichales;f__[Coprobacillaceae];Other | 0.0166534 | 0.0219127 |
| k__Bacteria;p__Firmicutes;c__Erysipelotrichi;o__Erysipelotrichales;f__[Coprobacillaceae];g__ | 0.5625562 | 4.8471383 |
| k__Bacteria;p__Firmicutes;c__Erysipelotrichi;o__Erysipelotrichales;f__[Coprobacillaceae];g__Coprobacillus | 1.1110576 | 0.0103910 |
| k__Bacteria;p__Proteobacteria;Other;Other;Other;Other | 0.0066196 | 0.0051955 |
| k__Bacteria;p__Proteobacteria;c__Alphaproteobacteria;Other;Other;Other | 0.0044005 | 0.0001834 |
| k__Bacteria;p__Proteobacteria;c__Alphaproteobacteria;o__;f__;g__ | 0.0116081 | 0.0000000 |
| k__Bacteria;p__Proteobacteria;c__Alphaproteobacteria;o__Caulobacterales;f__Caulobacteraceae;g__ | 0.0000759 | 0.0005501 |
| k__Bacteria;p__Proteobacteria;c__Alphaproteobacteria;o__Caulobacterales;f__Caulobacteraceae;g__Brevundimonas | 0.0000000 | 0.0003667 |
| k__Bacteria;p__Proteobacteria;c__Alphaproteobacteria;o__Caulobacterales;f__Caulobacteraceae;g__Caulobacter | 0.0000569 | 0.0000000 |
| k__Bacteria;p__Proteobacteria;c__Alphaproteobacteria;o__RF32;f__;g__ | 0.3223711 | 0.0014364 |
| k__Bacteria;p__Proteobacteria;c__Alphaproteobacteria;o__Rhizobiales;Other;Other | 0.0000000 | 0.0000917 |
| k__Bacteria;p__Proteobacteria;c__Alphaproteobacteria;o__Rhizobiales;f__Bradyrhizobiaceae;Other | 0.0000000 | 0.0000611 |
| k__Bacteria;p__Proteobacteria;c__Alphaproteobacteria;o__Rhizobiales;f__Bradyrhizobiaceae;g__Bradyrhizobium | 0.0000000 | 0.0007029 |
| k__Bacteria;p__Proteobacteria;c__Alphaproteobacteria;o__Rhizobiales;f__Brucellaceae;g__Ochrobactrum | 0.0015553 | 0.0000000 |
| k__Bacteria;p__Proteobacteria;c__Alphaproteobacteria;o__Rhizobiales;f__Methylobacteriaceae;g__ | 0.0004742 | 0.0000000 |
| k__Bacteria;p__Proteobacteria;c__Alphaproteobacteria;o__Rhizobiales;f__Methylobacteriaceae;g__Methylobacterium | 0.0001897 | 0.0000000 |
| k__Bacteria;p__Proteobacteria;c__Alphaproteobacteria;o__Rhizobiales;f__Methylocystaceae;g__ | 0.0001897 | 0.0000000 |
| k__Bacteria;p__Proteobacteria;c__Alphaproteobacteria;o__Rhizobiales;f__Phyllobacteriaceae;Other | 0.0000379 | 0.0000000 |
| k__Bacteria;p__Proteobacteria;c__Alphaproteobacteria;o__Rhizobiales;f__Phyllobacteriaceae;g__Phyllobacterium | 0.0000948 | 0.0135388 |
| k__Bacteria;p__Proteobacteria;c__Alphaproteobacteria;o__Rhizobiales;f__Xanthobacteraceae;g__Azorhizobium | 0.0000948 | 0.0000000 |
| k__Bacteria;p__Proteobacteria;c__Alphaproteobacteria;o__Rhodospirillales;f__Acetobacteraceae;g__ | 0.0001138 | 0.0000000 |
| k__Bacteria;p__Proteobacteria;c__Alphaproteobacteria;o__Rickettsiales;f__mitochondria;g__ | 0.0000000 | 0.0001222 |
| k__Bacteria;p__Proteobacteria;c__Alphaproteobacteria;o__Sphingomonadales;f__Erythrobacteraceae;Other | 0.0000000 | 0.0000917 |
| k__Bacteria;p__Proteobacteria;c__Alphaproteobacteria;o__Sphingomonadales;f__Sphingomonadaceae;Other | 0.0000759 | 0.0000000 |
| k__Bacteria;p__Proteobacteria;c__Alphaproteobacteria;o__Sphingomonadales;f__Sphingomonadaceae;g__Sphingomonas | 0.0027882 | 0.0000000 |
| k__Bacteria;p__Proteobacteria;c__Betaproteobacteria;Other;Other;Other | 0.0024278 | 0.0000306 |
| k__Bacteria;p__Proteobacteria;c__Betaproteobacteria;o__Burkholderiales;Other;Other | 0.2462926 | 0.0032090 |
| k__Bacteria;p__Proteobacteria;c__Betaproteobacteria;o__Burkholderiales;f__Alcaligenaceae;g__Sutterella | 0.0443270 | 0.0004584 |
| k__Bacteria;p__Proteobacteria;c__Betaproteobacteria;o__Burkholderiales;f__Comamonadaceae;Other | 0.0001517 | 0.0003056 |
| k__Bacteria;p__Proteobacteria;c__Betaproteobacteria;o__Burkholderiales;f__Comamonadaceae;g__Hydrogenophaga | 0.0000000 | 0.0001222 |
| k__Bacteria;p__Proteobacteria;c__Betaproteobacteria;o__Burkholderiales;f__Oxalobacteraceae;Other | 0.0013088 | 0.0000000 |
| k__Bacteria;p__Proteobacteria;c__Betaproteobacteria;o__Burkholderiales;f__Oxalobacteraceae;g__Herbaspirillum | 0.0000379 | 0.0000000 |
| k__Bacteria;p__Proteobacteria;c__Betaproteobacteria;o__Burkholderiales;f__Oxalobacteraceae;g__Oxalobacter | 0.0126703 | 0.0000611 |
| k__Bacteria;p__Proteobacteria;c__Betaproteobacteria;o__Burkholderiales;f__Oxalobacteraceae;g__Ralstonia | 0.0000569 | 0.0000000 |
| k__Bacteria;p__Proteobacteria;c__Betaproteobacteria;o__Neisseriales;f__Neisseriaceae;g__Neisseria | 0.0000000 | 0.0000000 |
| k__Bacteria;p__Proteobacteria;c__Deltaproteobacteria;Other;Other;Other | 0.0001517 | 0.0000000 |
| k__Bacteria;p__Proteobacteria;c__Deltaproteobacteria;o__Desulfovibrionales;Other;Other | 0.0051971 | 0.0000000 |
| k__Bacteria;p__Proteobacteria;c__Deltaproteobacteria;o__Desulfovibrionales;f__Desulfohalobiaceae;g__ | 0.0001138 | 0.0000000 |
| k__Bacteria;p__Proteobacteria;c__Deltaproteobacteria;o__Desulfovibrionales;f__Desulfovibrionaceae;Other | 0.0211108 | 0.0001528 |
| k__Bacteria;p__Proteobacteria;c__Deltaproteobacteria;o__Desulfovibrionales;f__Desulfovibrionaceae;g__Bilophila | 0.2951528 | 0.0014058 |
| k__Bacteria;p__Proteobacteria;c__Deltaproteobacteria;o__Desulfovibrionales;f__Desulfovibrionaceae;g__Desulfovibrio | 0.7463321 | 0.0032701 |
| k__Bacteria;p__Proteobacteria;c__Deltaproteobacteria;o__Myxococcales;Other;Other | 0.0000000 | 0.0000917 |
| k__Bacteria;p__Proteobacteria;c__Deltaproteobacteria;o__Myxococcales;f__;g__ | 0.0000759 | 0.0000000 |
| k__Bacteria;p__Proteobacteria;c__Epsilonproteobacteria;Other;Other;Other | 0.0001138 | 0.0000000 |
| k__Bacteria;p__Proteobacteria;c__Epsilonproteobacteria;o__Campylobacterales;Other;Other | 0.0000759 | 0.0000000 |
| k__Bacteria;p__Proteobacteria;c__Epsilonproteobacteria;o__Campylobacterales;f__Helicobacteraceae;Other | 0.0069800 | 0.0001222 |
| k__Bacteria;p__Proteobacteria;c__Epsilonproteobacteria;o__Campylobacterales;f__Helicobacteraceae;g__Flexispira | 0.0000379 | 0.0000000 |
| k__Bacteria;p__Proteobacteria;c__Epsilonproteobacteria;o__Campylobacterales;f__Helicobacteraceae;g__Helicobacter | 2.8778776 | 0.0146390 |
| k__Bacteria;p__Proteobacteria;c__Gammaproteobacteria;Other;Other;Other | 0.0001707 | 0.0198651 |
| k__Bacteria;p__Proteobacteria;c__Gammaproteobacteria;o__Alteromonadales;f__Pseudoalteromonadaceae;g__Pseudoalteromonas | 0.0000190 | 0.0000306 |
| k__Bacteria;p__Proteobacteria;c__Gammaproteobacteria;o__Enterobacteriales;f__Enterobacteriaceae;Other | 0.0009294 | 0.1879236 |
| k__Bacteria;p__Proteobacteria;c__Gammaproteobacteria;o__Enterobacteriales;f__Enterobacteriaceae;g__Erwinia | 0.0000000 | 0.0000611 |
| k__Bacteria;p__Proteobacteria;c__Gammaproteobacteria;o__Enterobacteriales;f__Enterobacteriaceae;g__Escherichia | 0.0084216 | 4.9540429 |
| k__Bacteria;p__Proteobacteria;c__Gammaproteobacteria;o__Oceanospirillales;f__Halomonadaceae;Other | 0.0000000 | 0.0000000 |
| k__Bacteria;p__Proteobacteria;c__Gammaproteobacteria;o__Pasteurellales;f__Pasteurellaceae;Other | 0.0007018 | 0.0719421 |
| k__Bacteria;p__Proteobacteria;c__Gammaproteobacteria;o__Pasteurellales;f__Pasteurellaceae;g__Aggregatibacter | 0.0000000 | 0.0020782 |
| k__Bacteria;p__Proteobacteria;c__Gammaproteobacteria;o__Pseudomonadales;f__Moraxellaceae;g__Acinetobacter | 0.0018209 | 0.0000611 |
| k__Bacteria;p__Proteobacteria;c__Gammaproteobacteria;o__Pseudomonadales;f__Pseudomonadaceae;g__Pseudomonas | 0.0000000 | 0.0000306 |
| k__Bacteria;p__Proteobacteria;c__Gammaproteobacteria;o__Xanthomonadales;f__Xanthomonadaceae;g__Stenotrophomonas | 0.0006639 | 0.0000000 |
| k__Bacteria;p__TM7;Other;Other;Other;Other | 0.0000000 | 0.0000000 |
| k__Bacteria;p__TM7;c__TM7-3;Other;Other;Other | 0.0000379 | 0.0000000 |
| k__Bacteria;p__TM7;c__TM7-3;o__CW040;f__F16;g__ | 0.0311825 | 0.0001222 |
| k__Bacteria;p__Tenericutes;c__Mollicutes;Other;Other;Other | 0.0009484 | 0.0005501 |
| k__Bacteria;p__Tenericutes;c__Mollicutes;o__Anaeroplasmatales;f__Anaeroplasmataceae;g__Anaeroplasma | 0.5772181 | 0.0068152 |
| k__Bacteria;p__Tenericutes;c__Mollicutes;o__Mycoplasmatales;f__Mycoplasmataceae;Other | 0.0679415 | 0.1206879 |
| k__Bacteria;p__Tenericutes;c__Mollicutes;o__Mycoplasmatales;f__Mycoplasmataceae;g__ | 0.0167103 | 0.0001222 |
| k__Bacteria;p__Tenericutes;c__Mollicutes;o__RF39;f__;g__ | 0.1431285 | 0.0007946 |
| k__Bacteria;p__Thermi;c__Deinococci;o__Thermales;f__Thermaceae;g__Meiothermus | 0.0000000 | 0.0000917 |
| k__Bacteria;p__Thermi;c__Deinococci;o__Thermales;f__Thermaceae;g__Thermus | 0.0001138 | 0.0000000 |
| k__Bacteria;p__Verrucomicrobia;Other;Other;Other;Other | 0.0000000 | 0.0006724 |
| k__Bacteria;p__Verrucomicrobia;c__Verrucomicrobiae;o__Verrucomicrobiales;f__Verrucomicrobiaceae;Other | 0.0000000 | 0.0005195 |
| k__Bacteria;p__Verrucomicrobia;c__Verrucomicrobiae;o__Verrucomicrobiales;f__Verrucomicrobiaceae;g__Akkermansia | 0.0215281 | 16.4645065 |
| k__Bacteria;Other;Other;Other;Other;Other | 1.3836012 | 0.9304188 |
| Unclassified;Other;Other;Other;Other;Other | 0.0001707 | 0.0003362 |
